# Roles of *Krüppel Homolog 1* and *Broad-Complex* in the Development of *Dendroctonus armandi* (Coleoptera: Scolytinae)

**DOI:** 10.3389/fphys.2022.865442

**Published:** 2022-04-06

**Authors:** Ya-Ya Sun, Dan-Yang Fu, Bin Liu, Lin-Jun Wang, Hui Chen

**Affiliations:** ^1^ State Key Laboratory for Conservation and Utilization of Subtropical Agro-bioresources, College of Forestry and Landscape Architecture, South China Agricultural University, Guangzhou, China; ^2^ College of Forestry, Northwest A&F University, Xianyang, China

**Keywords:** *Broad-complex*, *Dendroctonus armandi*, 20-hydroxyecdysone, juvenile hormone, *Krüppel homolog 1*, RNA interference

## Abstract

In insects, metamorphosis is controlled by juvenile hormone (JH) and 20-hydroxyecdysone (20E). *Krüppel homolog 1* (*Kr-h1*), a key JH-early inducible gene, is responsible for the suppression of metamorphosis and the regulation of the *Broad-Complex* (*Br-C*) gene, which is induced by 20E and functions as a “pupal specifier”. In this study, we identified and characterized the expression patterns and tissue distribution of *DaKr-h1* and *DaBr-C* at various developmental stages of *Dendroctonus armandi*. The expression of the two genes was induced by JH analog (JHA) methoprene and 20E, and their functions were investigated by RNA interference. *DaKr-h1* and *DaBr-C* were predominantly expressed in the heads of larvae and were significantly downregulated during the molting stage. In contrast, the *DaKr-h1* transcript level was highest in the adult anterior midgut. *DaBr-C* was mainly expressed in female adults, with the highest transcript levels in the ovaries. In the larval and pupal stages, both JHA and 20E significantly induced *DaKr-h1*, but only 20E significantly induced *DaBr-C*, indicating the importance of hormones in metamorphosis. *DaKr-h1* knockdown in larvae upregulated *DaBr-C* expression, resulting in precocious metamorphosis from larvae to pupae and the formation of miniature pupae. *DaKr-h1* knockdown in pupae suppressed *DaBr-C* expression, increased emergence, caused abnormal morphology, and caused the formation of small-winged adults. These results suggest that *DaKr-h1* is required for the metamorphosis of *D. armandi*. Our findings provide insight into the roles of *DaKr-h1* and *DaBr-C* in JH-induced transcriptional repression and highlight *DaKr-h1* as a potential target for metamorphosis suppression in *D. armandi*.

## Introduction

Insect metamorphosis, including larval–larval molting and larval–pupal–adult metamorphosis, is synergistically regulated by several insect hormones, most notably juvenile hormone (JH) and ecdysone (Riddiford, 1994; Riddiford et al., 2000). Juvenile hormone is secreted by the corpora allata in all insects from silverfish to *Drosophila* although their anatomical location differs slightly (Riddiford, 2012). It is considered a “status quo” hormone that maintains larval properties and inhibits metamorphosis during molting (Riddiford, 1996). 20-Hydroxyecdysone (20E), the active metabolite of ecdysone, induces larval–larval molting at high levels of JH, whereas it initiates larval–pupal and pupal–adult metamorphosis when the JH concentration drops sharply (Riddiford, 1994; Riddiford et al., 2010; Smykal et al., 2014; Daimon et al., 2015; Liu et al., 2018). Thus, the main function of JH is to prevent the premature metamorphosis (Riddiford, 1994). *Krüppel homolog 1* (*Kr-h1*), a C_2_H_2_ zinc finger transcription factor, plays an important role in the larval development of *Drosophila melanogaster* and *Tribolium castaneum*. Treatment with the JH analog pyriproxyfen during pupal development in *D. melanogaster* and *T. castaneum* resulted in *Kr-h1* upregulation and the formation of a “second pupa” rather than an adult. In the JH signaling pathway, *Kr-h1* is an important early responder gene (Pecasse et al., 2000; Minakuchi et al., 2008; Minakuchi et al., 2009; Zhu et al., 2010; Lozano and Belles, 2011; Zhang et al., 2011). *Kr-h1* homologs have been identified in several species, including *Apis mellifera* (Grozinger and Robinson, 2006), *Aedes aegypti* (Zhu et al., 2010), *Frankliniella occidentalis*, and *Haplothrips brevitubus* (Minakuchi et al., 2011).

The JH receptor Methoprene-tolerant regulates *Kr-h1*, which then regulates *Broad-Complex* (*Br-C*) expression (Abdou et al., 2011). The pupal specifier *Br-C* (Minakuchi, 2008; Minakuchi et al., 2009; Kayukawa et al., 2016) and the adult specifier Ecdysone-induced Protein 93F (*E93*) (Ureña et al., 2014; Ureña et al., 2016; Kayukawa et al., 2017) both rely on *Kr-h1* as a transcription repressor. *Br-C*, which is consisted of the Bric-a-brac–Tramtrack–Broad (BTB) complex and a zinc finger structure, is induced by 20E and functions as a “pupal specifier” during the larval–pupal transition (Kiss et al., 1976; Kiss et al., 1988; DiBello et al., 1991; Zhou and Riddiford, 2002). The structure and function of numerous *Br-C* genes have been characterized and analyzed from several insect species, including *Aedes aegypti, Blattella germanica*, *Bombyx mori*, *Drosophila melanogaster*, *Frankliniella occidentalis*, *Haplothrips brevitubus, Lymantria dispar* and *Manduca sexta* (Bayer et al., 1997; Zhou et al., 1998; Chen et al., 2004; Minakuchi et al., 2011; Yang et al., 2014; Ding et al., 2020)*.* In *D. melanogaster*, depletion of *DmKr-h1* with reduced *DmBr-C* levels in the anterior compartment and RNA interference (RNAi) affected larval pupation (Ureña et al., 2016). RNAi analysis in the *Blattella germanica* and *Pyrrochoris apterus* revealed that *Br-C* is specifically required for regulation of wing development, in particular size, shape and vein formation (Konopova et al., 2011; Huang et al., 2013). *Kr-h1* was also found to be induced by 20E. It was first postulated in the 1970s that 20E plays a molecular role in target cells during the larval–pupal transition. Gene expression analysis following 20E stimulation supports a model in which the ternary complex EcR/USP/20E activates transcription of *E75* and *Hr3*, which together control the delayed expression of *βFTZ-F1* (King-Jones and Thummel, 2005). Dynamic expression of *βFTZ-F1* is dependent on *Hr3* stimulation, and *Hr3* plays a key role in regulating the developmental switch by repressing 20E transcription of early response genes *E75, E74* and *Br-C* and activates the downstream late response factor *FTZ-F1* (White et al., 1997; Lam et al., 1999; Kageyama et al., 2003; Parvy et al., 2014).

RNA interference (RNAi) technology has now become a widely used tool to analyze the gene functions of Chinese white pine beetle (*Dendroctonus armandi* Tsai and Li). Chen‘s team used RNAi to investigate and analyze the olfactory receptor coreceptor (*DarmOrco*), chemoreceptor (*DarmCSP2*), aquaporins (*DaAqps*), antifreeze protein genes (*DaAFP*), Capa peptide receptors (*DaCapaRs*), neuropeptide F (*DaNPF*), and 3-hydroxy-3-methylglutaryl coenzyme A reductase genes (*HMGR*) in *D. armandi* (Zhang et al., 2016; Li et al., 2018; Fu et al., 2019, 2020, 2021; Liu et al., 2021; Sun et al., 2021). Sun et al. (2022) discovered that allatostatin C (PISCF/AST) and juvenile hormone acid O-methyltransferase (*JHAMT*) were major regulators of juvenile hormone synthesis in *D. armandi* after obtaining dsRNA technology via the L4440 vector construction.

RNAi technique was used to characterize the activities of two genes in *D. armandi, DaKr-h1* and *DaBr-C*. Their expression induced by JHA and 20E were analyzed in different tissues at different developmental stages by a series of RNAi experiments. While JH may prevent premature larval–adult metamorphosis by direct *Kr-h1*-dependent *Br-C* gene repression, JH-induced transcriptional repression of the target genes leads to the emergence of supernumerary pupae during the pupal–adult transition. Furthermore, ingesting bacterially generated dsRNA could be an effective RNAi-based method for controlling insect pests.

## Materials and Methods

### Insects

We collected *Pinus armandii* Franch infested with *D. armandi* on the southern slopes of central Qinling Mountains (33°18 ′–33°28′ N, 108°21 ′–108°39′ E) in Shaanxi, China, and placed the specimens in a greenhouse. The adult insects were collected after they emerged and stored on moist paper at 4°C. The sex of adults was based on external genitalia and male-specific auditory cues (Dai et al., 2014; Zhao et al., 2017). Larvae and pupae were collected from under the bark of infected *P. armandii.*

### Ribonucleic Acid Isolation and cDNA Synthesis

Total RNA was isolated from three beetles by the UNIQ-10 Column Trizol Total RNA Isolation Kit (Sangon Biotech, Shanghai, China) in accordance with the manufacturer’s protocol. Its integrity was checked on 1% agarose gels and quantified using NANO DROP 2000 spectrophotometry (Thermo Scientific, Pittsburgh, Pennsylvania, United States of America). The purity was calculated by mean of relation A260/A280 ratio (μg/mL = A260 × dilution factor × 40). The synthesized cDNA obtained from the sample was used as the template using the TransScript One-Step gDNA Removal and cDNA Synthesis SuperMix (TransGen Biotech, Beijing, China).

### Amplification of Genes, Cloning and Sequence Analyses

cDNA synthesized from the sample was used as a template for PCR reaction. In Primer Premier 5.0, specific primers (Supplementary Table S1) were designed based on *Kr-h1* and *Br-C* sequences of *Dendroctonus ponderosae* from NCBI (http://www.ncbi.nlm.nih.gov/). PCR amplifications were performed in a C1000 thermocycler (Bio-Rad, Hercules, CA, United States), cDNA amplification was performed in a 20 μL reaction volume: 1 μL cDNA, 0.25 μM each primer, 10 μL EcoTaq PCR SuperMix (TransGen Biotech, Beijing, China), with ddH_2_O added to 20 μL. The reaction conditions were as follows: 94°C for 5 min, 30 cycles of 94°C for 30 s, TM of each pair of primers for 30 s and 72°C for 30 s with a final extension for 10 min at 72°C for 30 s. The PCR products were visualized on 1% agarose gels stained with 1× DuRed and compared with a 2 K plus DNA marker (TransGen Biotech, Beijing, China).

Single-stranded 5and 3′ RACE-ready cDNA was synthesized from RNA using a SMARTer RACE cDNA Amplification Kit (Clontech Laboratories Inc., Mountain, CA, United States) according to the manufacturer’s protocol. Partial sequences were used in the primer design, and the PCR was performed as described in the SMARTer™ RACE cDNA Amplification Kit (Clontech Laboratories Inc., Mountain, CA, United States). The amplicons were purified, cloned and sequenced. Sequences were manually edited with EditSeq from DNASTAR (https://www.dnastar.com/) to obtain inserts, which were then BLASTed against the NCBI database. The complete sequences were compared using a BlastP search with those deposited in GenBank (Altschul et al., 1990).

### Sequence Analyses of the Genes

The molecular mass (kDa) and isoelectric point (IP) of the two sequences were determined by the ProtParam program (Gasteiger et al., 2005). *Kr-h1* and *Br-C* of *D. armandi* were checked for likely subcellular localization using Target P1.1 software (http://www.cbs.dtu.dk/services/TargetP/) with the default parameters (Emanuelsson et al., 2000).

In order to identify *Kr-h1 and Br-C* in *D. armandi*, a phylogenetic inference analysis of 11 full-length sequences was performed by the neighbor-joining method with MEGA7.0 (Le and Gascuel, 2008; Kumar et al., 2016). To estimate the support for each node, bootstrap values were calculated after 1,000 pseudoreplicates.

### Analysis of the *DaKr-h1* and *DaBr-C* Genes Transcript Levels (Real Time-qPCR)

#### Expression Patterns of Different Life Stages and Tissues

During development, *D. armandi* larvae were separated into three sub-stages: small larvae (SL: penultimate (or pre-final) instar larva weighing less than 2.5 mg); large larvae (LL: final instar, feeding larva weighing 5.0–7.0 mg); mature larva (ML: post-feeding final instar larva). Pupae were separated into five sub-stages: P0: pupae, P1: Day 1 of the pupal stage, P2: Day 2 of the pupal stage, P3: Day 3 of the pupal stage and P4: Day 4 of the pupal stage. *D. armandi* adults were separated into four sub-stages: teneral adults (TA: body color still light), dark brown adults (DbA: Adults darkened to dark brown, but were still under the bark and had not migrated), emergent adults (EA), and feeding adults (invading a new host). The difference between the teneral adults and dark brown adults is the difference in body color. The difference between dark brown and emergent is that the former is dark brown and still under the bark, while the emergent adults are black and have already emerged from the bark. The difference between emergent adults and feeding adults is that emergent adults is when the insect has just emerged from the bark and emerged, while new feeding adults is when the insect has emerged and invaded a new host and fed. There were three biological replicates per developmental stage, each containing three insects (Dai et al., 2014).

In terms of tissue distribution, for tissue-specific analysis of *DaKr-h1*and *DaBr-C* genes, 60 males and 60 females that had emerged as adults (head, anterior midgut, hindgut, Malpighian tubule, fat body, reproductive organ (testes of males and ovaries of females) and antennae), 30 larvae and 30 pupae (head, gut, fat body, *epidermis*) were dissected, frozen immediately in liquid nitrogen and stored at -80°C. Each tissue was replicated three times, and a pool of total RNA extracted from different tissues was used per replicate. RNA isolation and cDNA synthesis followed the protocols described above.

#### Effects of JH Analog Injection on Transcript Levels of *DaKr-h1* and *DaBr-C*

Solutions of the stock juvenile hormone analog JHA methoprene (Sigma, Saint Louis, United States), were separately diluted to 5, 25 and 100 μg/μL concentrations using acetone (Huang et al., 2016). Next, 0.1 μL of each JHA dilution was injected into *D. armandi* larvae (mature larvae) and pupae (newly pupated pupae) through the ventral abdomen using Hamilton Microliter syringes (700 series, RN) with 32G sharp-point needles (Hamilton, Switzerland) to a final JHA content of 0.5, 2.5 or 10 μg. Meanwhile, an equal amount of acetone was injected as the solvent control. To analyze the expression of the JH-induced genes, the total RNA was extracted after 0, 24, 48 and 72 h of JHA or acetone treatment and subjected to cDNA synthesis and qRT-PCR.

#### Effects of 20E Injection on Transcript Levels of *DaKr-h1* and *DaBr-C*

Solutions of the stock Ecdysterone (20E, 20-Hydroxyecdysone; Sangon Biotech, Shanghai, China) were separately diluted to 5, 25 and 100 μg/μL concentrations using ethanol. Next, 0.1 μL of each 20E dilution was injected into *D. armandi* larvae (mature larvae) and pupae (newly pupated pupae) through the ventral abdomen using Hamilton Microliter syringes (700 series, RN) with 32G sharp-point needles (Hamilton, Switzerland) to a final 20E content of 0.5, 2.5 or 10 μg. Meanwhile, an equal amount of ethanol was injected as the solvent control. To analyze the expression of the 20E-induced genes, the total RNA was extracted after 0, 24, 48 and 72 h of 20E or ethanol treatment and subjected to cDNA synthesis and qRT-PCR. Three biological replicates were measured, each containing three beetles.

### dsRNA Synthesis

#### Target Genes

The *Krüppel homolog 1* (*Kr-h1*) and *Broad-Complex* (*Br-C*) genes of *D. armandi* were identified in 2.3 above. The sequences of *DaKr-h1 and DaBr-C* genes were digested with *Xba*I and *Sma*I. The *DaKr-h1* sequences were amplified with primers (Supplementary Table S1) using EcoTaq PCR SuperMix (TransGen Biotech, Beijing, China) and a C1000 thermo cycler (Bio-Rad, Hercules, CA, United States). The polymerase chain reaction (PCR) amplification reaction conditions were as mentioned earlier.

#### Vector Construction and Expression

##### Construction of Transformed *E. Coli* Expressing dsRNA

PCR products obtained in the previous steps were digested and cloned into the plasmid vector, L4440 (Wuhan Miaoling Biotechnology Co., Ltd., Wuhan, China), between the *Xba*I and *Sma*I restriction sites. Successful cloning was verified through PCR and sequencing. Plasmids containing the correct insert were extracted and transformed into *E. coli* strain HT115 (DE3) strain (Shanghai Weidi Biotechnology Co., Ltd., Shanghai, China). Positive clones were incubated at 37°C until the mid-exponential phase (OD600 = 0.4). To activate the T7 promoter for RNA transcription, IPTG (isopropyl-β-D-1-thiogalactopyranoside) was added to a final concentration of 0.8 mM and then incubated for an additional 4 h under the same conditions. Each bacterial cultures (100 ml) was transferred into a 50-ml Falcon tube and centrifuged at 4,000×*g* for 10 min at 4°C.

##### Isolation of dsRNA Using Conventional Method

Cells were harvested via centrifugation at 4,000 *g* and 4°C for 10 min. Bacteria were 10× concentrated and split into two vials. One vial (1 ml cell suspension) was used to extract RNA by UNIQ-10 Column Trizol Total RNA Isolation Kit (Sangon Biotech, Shanghai, China) according to the manufacturer’s protocol. The extracted RNA was compared with the dsRNA not induced by IPTG to determine whether IPTG had been successfully induced. Its integrity was checked on 1% agarose gels, and quantification was performed by spectrophotometry with a NANO DROP 2000 (Thermo Scientific, Pittsburgh, Pennsylvania, United States). The successfully induced vial were centrifuged at 4°C, 4,000 × *g* for 10 min. The supernatant was discarded, 500 μL of Trizol was added to bacterial pellet, and total RNA was extracted and subjected to DNAse treatment. The reactions were allowed to proceed overnight at 42°C, followed by both the RNase and DNase digestion and purification steps to obtain the dsRNA. The dsRNA was spectrophotometrically quantified before injection.

#### RNAi Experiment

Synthesized dsRNA (0.2 μL) were injected into the ventral abdomen of the larvae on the first day of the last instar or pupae using a 10 μL Hamilton Microliter syringes (700 series, RN) with 32G sharp-point needles (Hamilton, Switzerland). dsRNA of L4440-Kr-h1 and L4440-Br-C (IPTG was not added) were used as negative controls. Untreated beetles were used as blank controls. Each beetle was injected only once. For each dose, three of the treated beetles were randomly selected at 24 and 72 h, frozen immediately in liquid nitrogen and stored at −80°C. The expression levels of *DaKr-h1 and DaBr-C* were quantified first, and the expression of *DaBr-C* was quantified only in beetles, which *DaKr-h1* were successfully knocked down. Twenty-five larvae were observed and the survival rate was recorded, and 25 pupae were observed for defective wings after plumentation and repeated three times.

### Real-Time Polymerase Chain Reaction

Specific qRT-PCR primers were designed by Primer Premier 5.0 on the basis of the obtained nucleotide sequences (Supplementary Table S1). The melting curve analysis was performed to ensure that only a single product corresponding to the target sequence was amplified. All primer pairs were tested in advance to obtain close to 100%. The expression of the *CYP4G55* (Dai et al., 2015) and *β-actin* (Dai et al., 2014) genes was used as an internal control. Real-time PCR was performed in triplicate according to the manufacturer’s instructions using TransStart Top Green qPCR SuperMix (TransGen Biotech, Beijing, China) on a CFX96TM Real-Time qPCR Detection System (Bio-Rad, Hercules, California, United States). The qPCR was performed using the following program: 95 °C for 10 min; 40 cycles at 95°C for 5 s, TM of each pair of primers (Supplementary Table S1) for 15 s and 72°C for 20 s.

### Statistics

The 2^−ΔΔCt^ method was used to determine the effect of interference. According to a role of thumb, transcript levels below 0.5 relative to the control were considered to indicate a significant effect of RNAi. One-way analysis of variance (ANOVA) (*p* < 0.05) and two-way analysis was used to determine significance of different treatments. For gene silencing analysis, an unpaired *t*-test was used to compare differences of two groups. The Kaplan-Meier method (log rank (Mantel–Cox)) was used to analyze the survival rates (*p* < 0.05) (Gillespie and Fisher, 1979). All statistical analyses were performed using SPSS Statistics 21.0 (IBM, California, IL, United States) and plotted using Prism 5.0 (GraphPad Software, CA, United States).

## Results

### Identification of *DaKr-h1* and *DaBr-C* Genes

*DaKr-h1* and *DaBr-C* were identified from *D. armandi*, and the full-length amino acid sequences shared the highest identity (96.13–98.35%) with *D. ponderosae* (Table 1, Supplementary Figure S1). From the obtained values, *DaKr-h1* and *DaBr-C* were assigned as corresponding homologs of *D. ponderosae Kr-h1* and *Br-C*.

**TABLE 1:** Amino acid identity of putative *DaKr-h1* and *DaBr-C* with related sequences in other insect species.

Genes	Blast Matches in Gene Bank			Identity in the Full length^a^
	Species	Gene	Accession No	Blastp (%)
*DaKr-h1*	*Dendroctonus ponderosae*	*Kr-h1*	XP_019,756,355.1	96.13
	*Sitophilus oryzae*	*Kr-h1*	XP_030,765,511.1	79.00
	*Anoplophora glabripennis*	*Kr-h1*	XP_018,575,408.1	67.85
*DaBr-C*	*Dendroctonus ponderosae*	*Br-C*	XP_019,758,737.1	98.35
	*Sitophilus oryzae*	*Br-C*	XP_030,752,056.1	88.41
	*Anoplophora glabripennis*	*Br-C*	XP_018,566,180.1	75.00

a As predicted by BLAST (www.ncbi.nlm.nih.gov) (Altschul et al., 1990).

**FIGURE 1:**
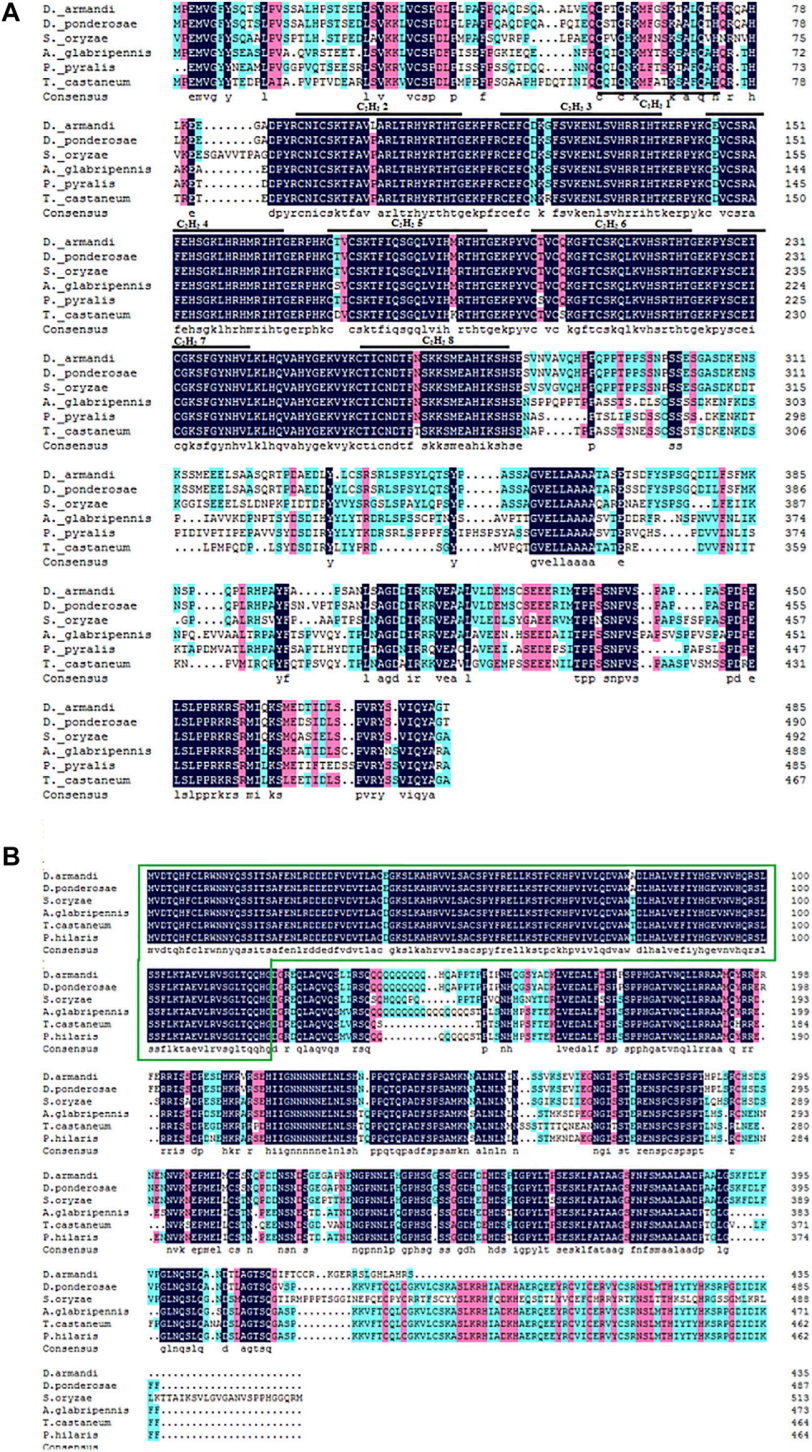
Structures of *DaKr-h1* and *DaBr-C* in *D. armandi*. **(A)** Alignment of the putative *DaKr-h1* sequence in beetle species and their consensus sequences with *D. armandi* identified sequences. Black lines represent zinc finger structures C_2_H_2_ 1–8, marked with numbers 1–8, respectively. **(B)** Alignment of the *DaBr-C* sequence. The green box represents the Bric-a-brac–Tramtrack–Broad complex.

Analysis of the deduced amino acid sequences of *DaKr-h1* revealed the presence of eight adjacent Cys_2_/His_2_ zinc finger DNA-binding domains numbered from Z1 to Z8. These zinc finger regions, often called CysX_2_CysX_12_HisX_3_His, indicate the spacers between the zinc-binding residues (Duportets et al., 2012) (Figure 1A). *DaBr-C*, an insect-specific transcription factor, has a BTB structural domain, which is a protein–protein interaction motif, at the N-terminus (Zhou et al., 1998) (Figure 1B). The full-length open reading frames (ORFs) of *DaKr-h1* and *DaBr-C* were 1460 bp and 1305 bp, encoding 491 and 434 amino acids. Respectively, the predicted molecular mass were 53.90 and 47.75 kDa, and the isoelectric point were 8.71 and 5.80; Target P 1.1 program the predicted subcellular location of *DaKr-h1* and *DaBr-C* suggest cytoplasmic location (Table 2).

**TABLE 2:** Physicochemical properties and cellular localization of *DaKr-h1* and *DaBr-C* of *D. armandi*.

Gene Name	ORF Size (Aa/Bp)^a^	*M*w (kDa)^a^	I.P^a^	Signal Peptide Prediction^b^
*DaKr-h1*	491/1,460	53.90	8.71	SP 0.9988 mTP 0.001 other 0.0001
*DaBr-C*	434/1,305	47.75	5.80	SP 1.0000 mTP 0.000 other 0.0000

a As predicted by the ProtParam program (Gasteiger et al., 2005).

b As predicted by Target *p* 1.1 program (Emanuelsson et al., 2000).

I.P.: isoelectric point; *M*w: molecular weight; ORF: open reading frame; SP: secretory pathway signal peptide; mTP: mitochondrial targeting peptide.

### *DaKr-h1* and *DaBr-C* Transcript Levels in Different Tissues of *D. armandi* at Different Life Stages

**FIGURE 2:**
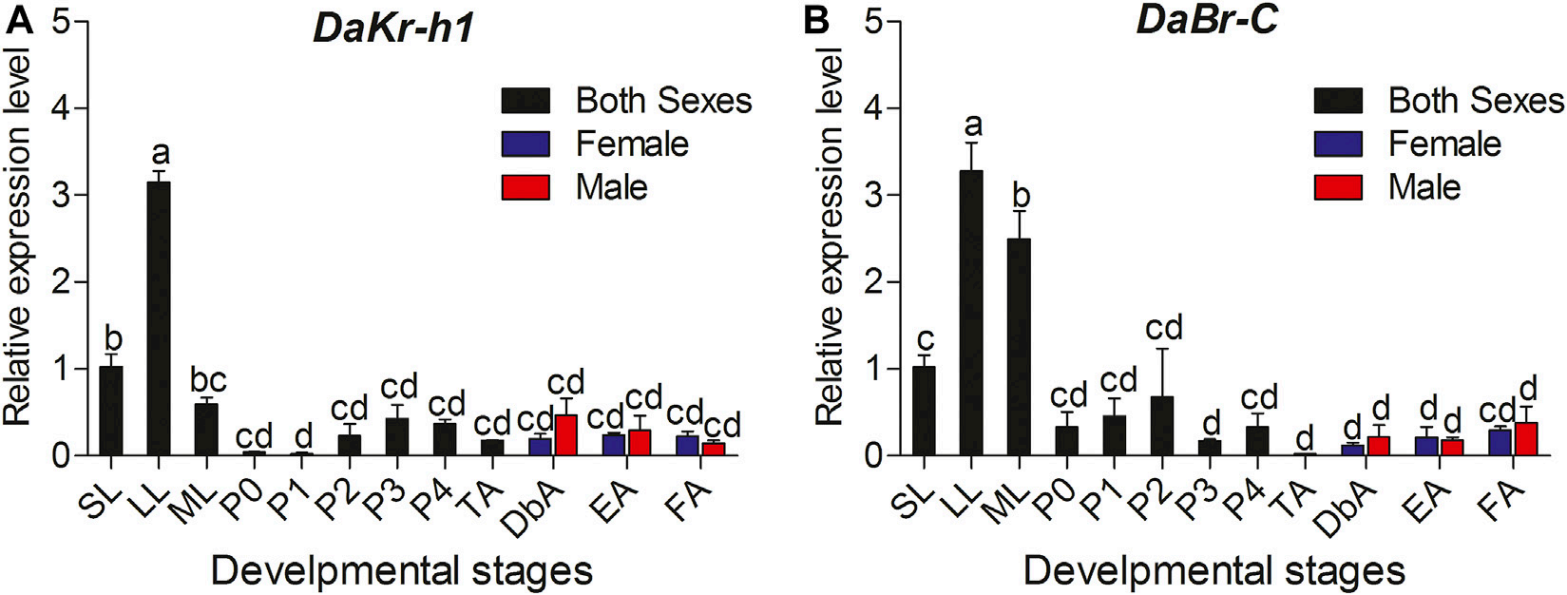
Quantitative expressions of *DaKr-h1* and *DaBr-C* genes at different stages of *D. armandi*. **(A)** *DaKr-h1*; **(B)** *DaBr-C*. Small larvae (SL: penultimate (or pre-final) instar larva weighing less than 2.5 mg); large larvae (LL: final instar, feeding larva weighing 5.0–7.0 mg); mature larva (ML: post-feeding final instar larva). Pupae were separated into five sub-stages: P0: pupae, P1: Day 1 of the pupal stage, P2: Day 2 of the pupal stage, P3: Day 3 of the pupal stage and P4: Day 4 of the pupal stage. *D. armandi* adults were separated into four sub-stages: teneral adults (TA: body color still light), dark brown adults (DbA: Adults darkened to dark brown, but were still under the bark and had not migrated), emergent adults (EA), and feeding adults (invading a new host) of *D. armandi*. Data are means ± SE of three independent experiments. All templates were normalized with *CYP*4G55 and *β-actin*. The 2^−ΔΔCt^ and SE values were used for plotting the graph. Different letters indicate significant differences at *p* < 0.05 (one-way ANOVA, with Tukey’s test of multiple comparisons).

Transcript levels were measured by qRT-PCR. Relative to the larval stage, one-way ANOVA showed statistically significant differences in transcript levels among the developmental stages (*DaKr-h1*: F statistic (F) = 6.128, degree of freedom (df) = 14, significance level (*p*) < 0.0001; *DaBr-C*: F = 2.402, df = 14, *p* = 0.022). *DaKr-h1* and *DaBr-C* expression tended to increase from the small larval stage, reached the highest value in the large larval stage, decreased in the mature larval stage, and remained stable from the pupal to adult stage. No significant difference was observed in the expression between males and females (Figure 2).

**FIGURE 3:**
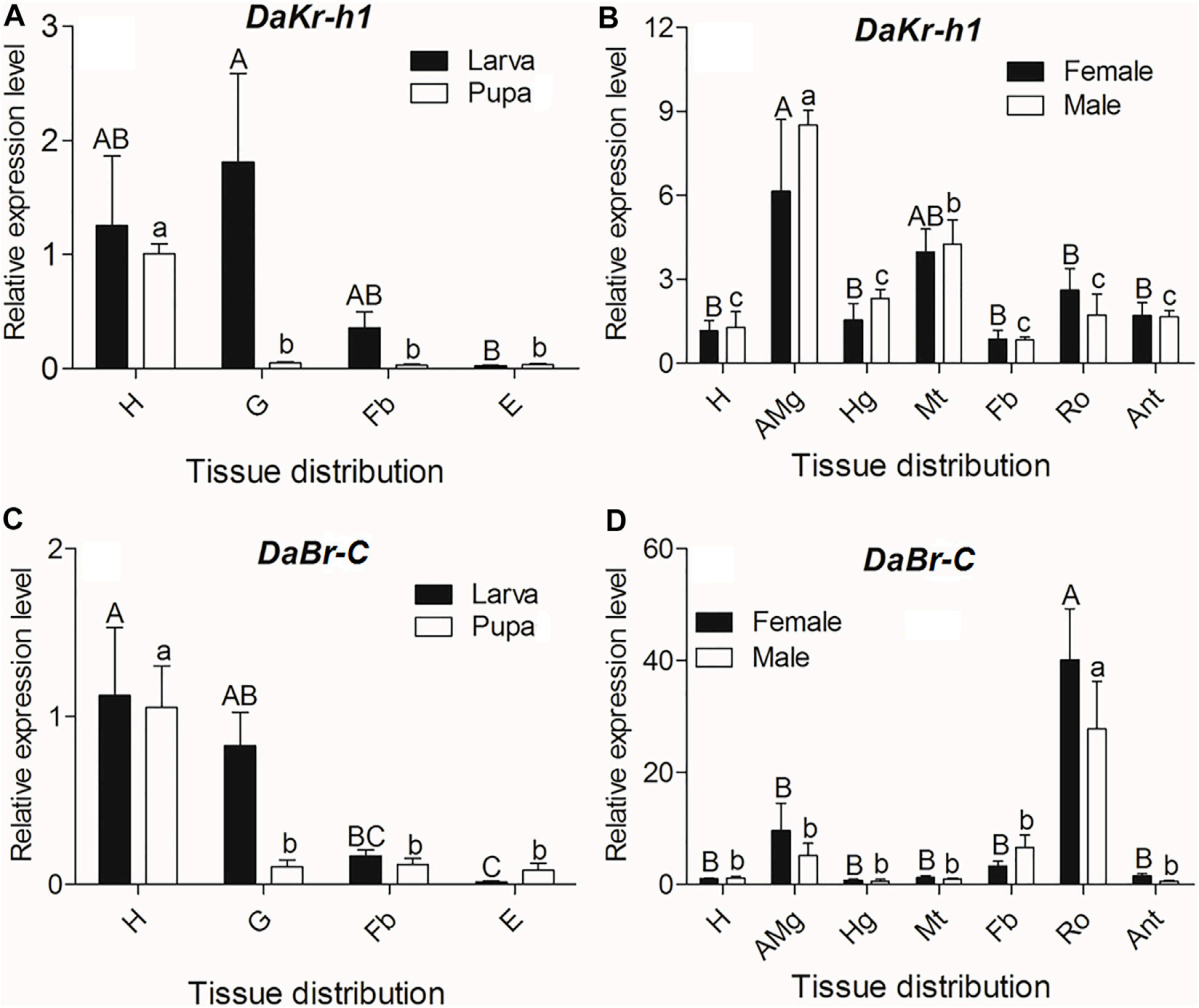
Quantitative expressions of *DaKr-h1* and *DaBr-C* genes (means ± SE) in different tissues of larva, pupa, and emerged adult. The tissues include head (H), anterior midgut (AMg), hindgut (Hg), Malpighian tubule (Mt), fat body (Fb), reproductive organs (Ro: testes of males and ovaries of females), antennae (Ant), gut (G), and *epidermis* (E) of *D. armandi*. Data are means ± SE of three independent experiments. In both graphs, transcript levels are shown relative to the head (H) of males and females. All templates were normalized with *CYP4G55* and *β-actin*. The 2^−ΔΔCt^ and SE values were used for plotting the graphs. Uppercase letters indicate significant differences of females or larvae, whereas lowercase letters indicate significant differences of males or pupae.

To understand the functional roles of *DaKr-h1* and *DaBr-C*, we studied the tissue-specific expression in three developmental stages (i.e., larva, pupa, and adult). qRT-PCR analysis showed statistically significant differences between different tissues at all developmental stages. In larvae, the *DaKr-h1* and *DaBr-C* transcript levels were highly expressed in the head and gut (*DaKr-h1*: F = 4.386, df = 3, *p* = 0.042; *DaBr-C*: F = 4.386, df = 3, *p* = 0.042). *DaKr-h1* and *DaBr-C* were highly expressed in the head of pupae (*DaKr-h1*: F = 15.022, df = 3, *p* = 0.001; *DaBr-C*: F = 15.022, df = 3, *p* = 0.001) (Figures 3A,C). In adults, the *DaKr-h1* transcript level was higher in the midgut and in the Malpighian tubules, than in the head, hindgut, fat body, testes, ovaries, and antennae (Figure 3B). Whereas, the *DaBr-C* transcript level was highest in the ovaries of females and testes of males (F = 15.369, df = 6, *p* < 0.0001) (Figure 3D).

Thus, the *DaKr-h1* gene exhibited a broad tissue expression pattern, reflecting the possible pleiotropic action of *Kr-h1* in *D. armandi*.

### Effects of JH Analog Injection on *DaKr-h1* and *DaBr-C* Transcript Levels

**FIGURE 4:**
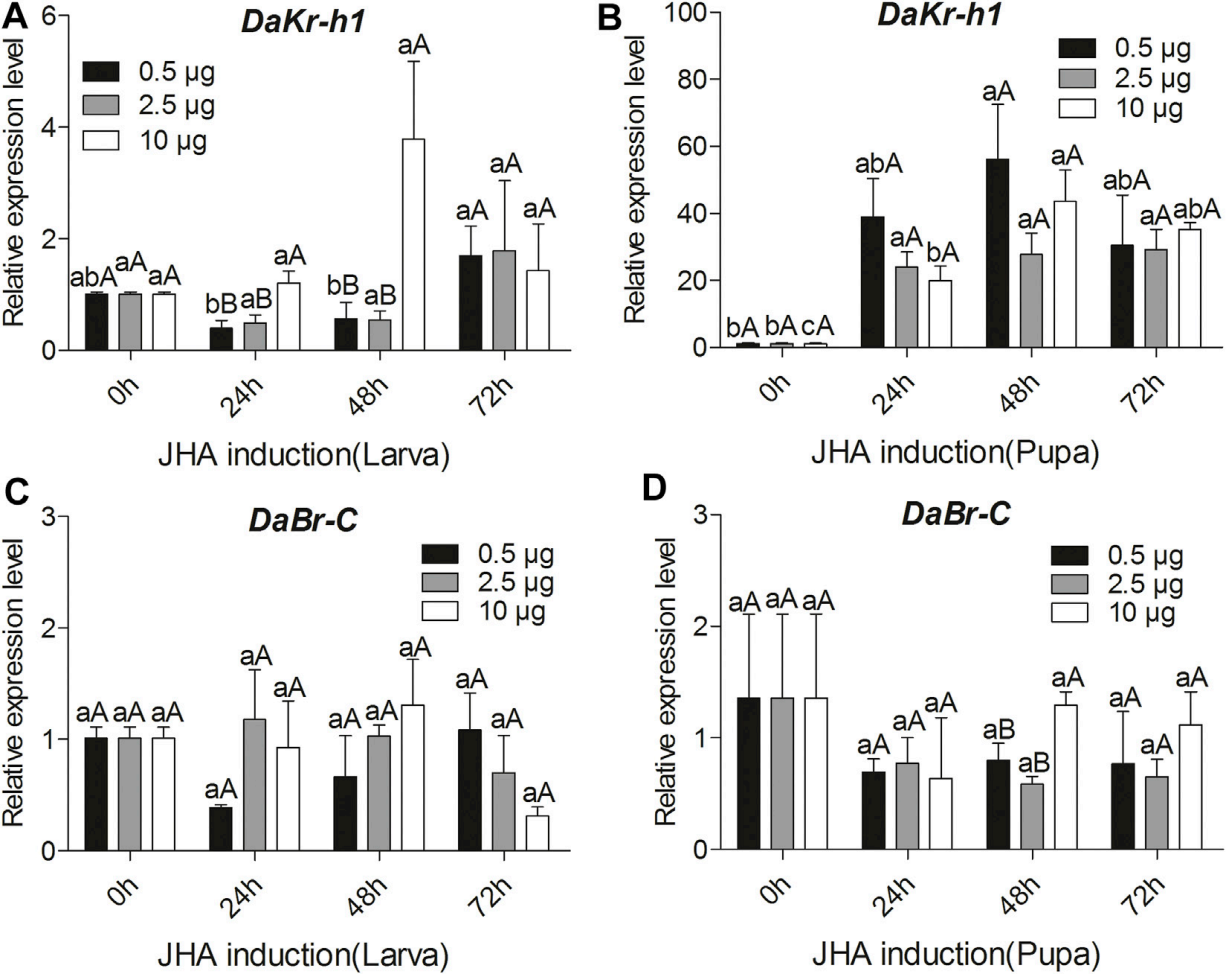
Quantitative expressions of *DaKr-h1* and *DaBr-C* genes with JHA (0.5, 2.5, 10 μg) at different larval and pupal stages. Transcript levels of **(A)** *DaKr-h1* in larvae, **(B)** *DaKr-h1* in pupae, **(C)** *DaBr-C* in larvae, and **(D)** *DaBr-C* in pupae. Data are means ± SE of three independent experiments. In all graphs, transcript levels are shown relative to acetone-treated larvae and pupae (control). All templates were normalized with *CYP4G55* and *β-actin*. The 2^−ΔΔCt^ and SE values were used for plotting the graphs. Different letters indicate significant differences at *p* < 0.05 (two-way ANOVA). Uppercase letters indicate significant differences of concentration at the same time, whereas lowercase letters indicate significant differences of time at the same concentration.

To reveal the molecular mechanism of the influence of JHA on *DaKr-h1* expression, the relative expression profile of *DaKr-h1* was analyzed by qRT-PCR at more time points after JHA treatment. qRT-PCR analysis showed that low level of methoprene (0.5 µg) suppress *DaKr-h1* expression for the first 48 h after injection in the larva, then it returns to normal levels at 72 h. By contrast, 10 µg methoprene induces it by 48 h after which it declines back to normal, likely indicating that the methoprene has been metabolized or excreted by that time (Figure 4A). In the pupa Figure 4B shows clearly that all doses of the JH analog induce *DaKr-h1* mRNA with the lower doses being more effective earlier (24 h). Similarly, for *DaBr-C* expression, methoprene treatment had no effect on broad expression in the larva but in the pupa the lower doses appeared to depress *DaBr-C* expression at 48 h (Figures 4C,D).

### Effects of 20E Injection on *DaKr-h1* and *DaBr-C* Transcript Levels

**FIGURE 5:**
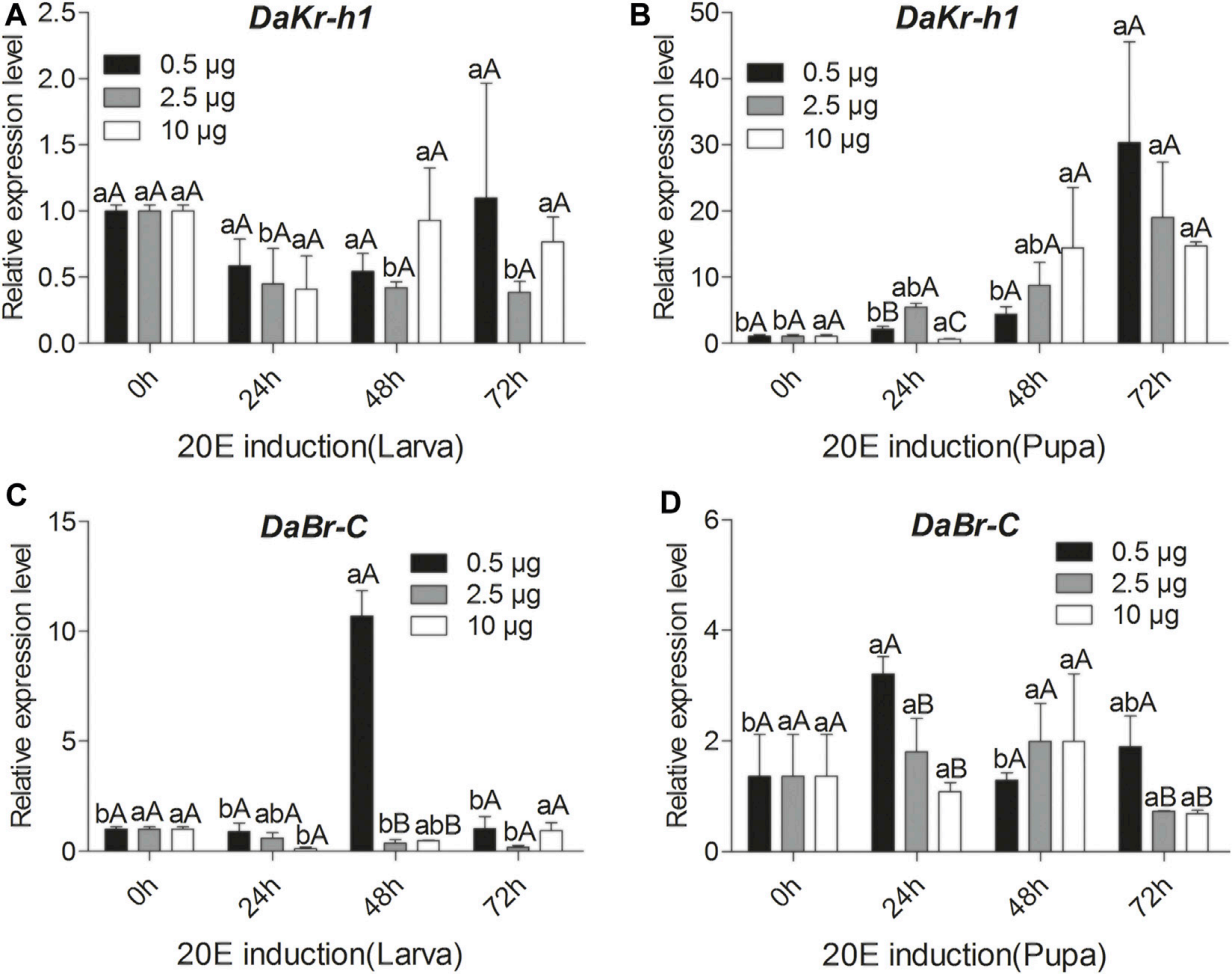
Quantitative expressions of *DaKr-h1* and *DaBr-C* genes with the ecdysteroid 20-hydroxyecdysone (20E; 0.5, 2.5, 10 μg) at different larval and pupal stages. Transcript levels of **(A)** *DaKr-h1* in larvae, **(B)** *DaKr-h1* in pupae, **(C)** *DaBr-C* in larvae, and **(D)** *DaBr-C* in pupae. Data are means ± SE of three independent experiments. In both graphs, transcript levels are shown relative to ethanol-treated larvae and pupae (control). All templates were normalized with *CYP4G55* and *β-actin*. The 2^−ΔΔCt^ and SE values were used for plotting the graphs. Different letters indicate significant differences at *p* < 0.05 (two-way ANOVA). Uppercase letters indicate significant differences of concentration at the same time, whereas lowercase letters indicate significant differences of time at the same concentration.

qRT-PCR analysis showed that 2.5 µg of 20E suppress *DaKr-h1* expression for 72 h after injection in the larvae but other dose (0.5 and 10 µg) of 20E treatment had no effect on *DaKr-h1*expression in the larva (Figure 5A). In the pupa, Figure 5B clearly shows that low levels of 20E (0.5, 2.5 µg) induced *DaKr-h1* expression by 72 h after injection. Also, low levels of 20E (0.5 µg) induced *DaBr-C* expression only at 48 h after the larval injection. By contrast, 2.5 and 10 µg of 20E suppress *DaBr-C* expression for 48 h, after which it rose back to normal levels. In the pupa, low level of 20E (0.5 µg) induced *DaBr-C* expression during the first 24 h after injection, and then returned to normal levels within 72 h. However, other dose (2.5 and 10 µg) of 20E treatment had no effect on the broad expression of pupa (Figures 5C,D).

### dsRNA Expression

**FIGURE 6:**
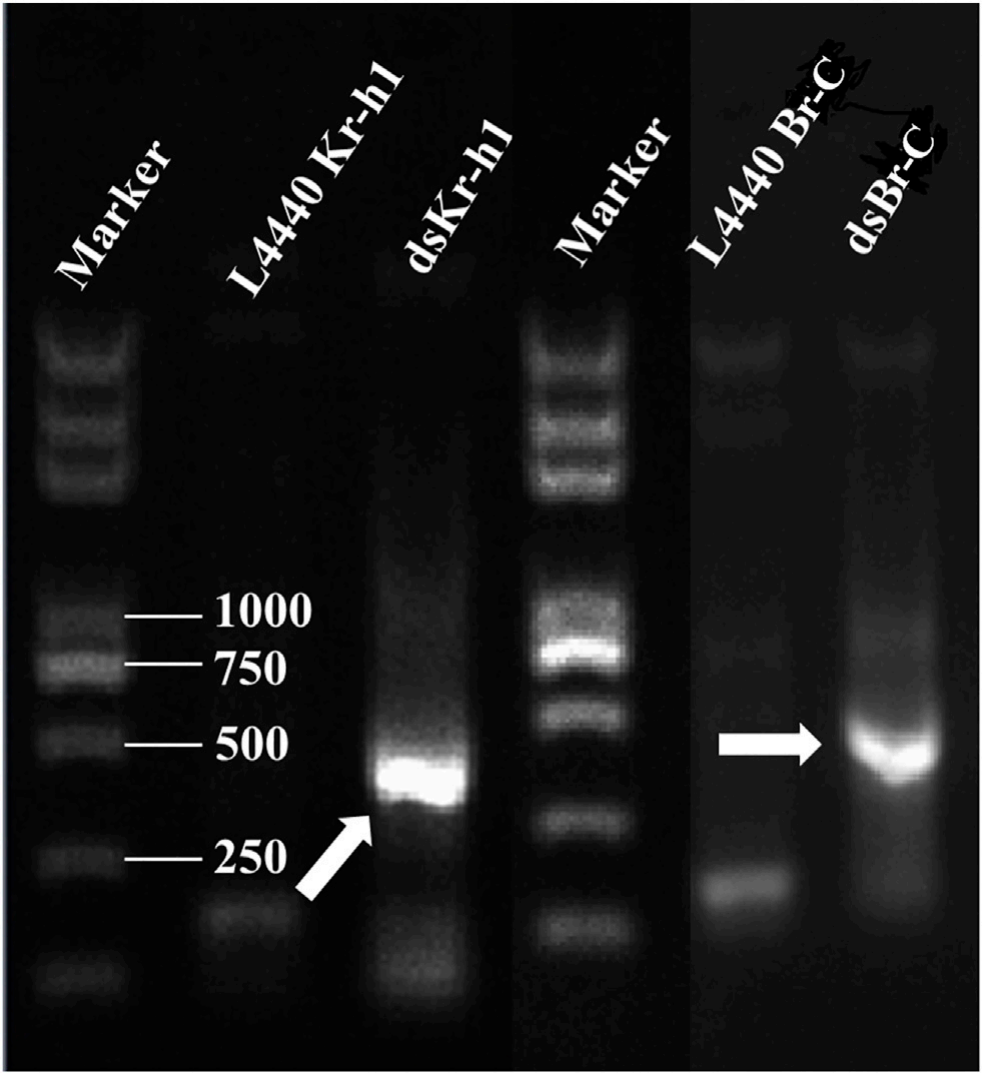
Confirmation of dsRNA produced in HT115 cells. The recombinant plasmids were transformed into HT115 competent cells. Individual transformants were cultured on 2 × yeast–tryptone media with addition of isopropyl β-d-1-thiogalactopyranoside. The cell cultures were processed for total RNA extraction. Lane M: 2 kb Plus DNA marker (TransGen Biotech, Beijing, China). Arrowhead indicates the position of the dsRNA band.

Two expression vectors L4440–*Kr-h1* and L4440–*Br-C* corresponding to *DaKr-h1* and *DaBr-C* were constructed on the basis of the L4440 vector. The plasmid was digested with restriction endonucleases *Xba*I and *Sma*I, and gel electrophoresis showed that one line was about 390 bp from L4440–*Kr-h1* and the other 489 bp from L4440–*Br-C.* After HT115-carrying plasmids L4440–*Kr-h1* and L4440–*Br-C* were induced by IPTG, the total RNA (containing ds*Kr-h1* or ds*Br-C*) was extracted from engineered bacteria. Gel electrophoresis showed that the residual RNA were ds*Kr-h1* and ds*Br-C* bands (Figure 6).

### Effects of RNAi on *DaKr-h1* and *DaBr-C* Expression

#### Determination of *DaKr-h1* and *DaBr-C* Silencing by qRT-Polymerase Chain Reaction

**FIGURE 7:**
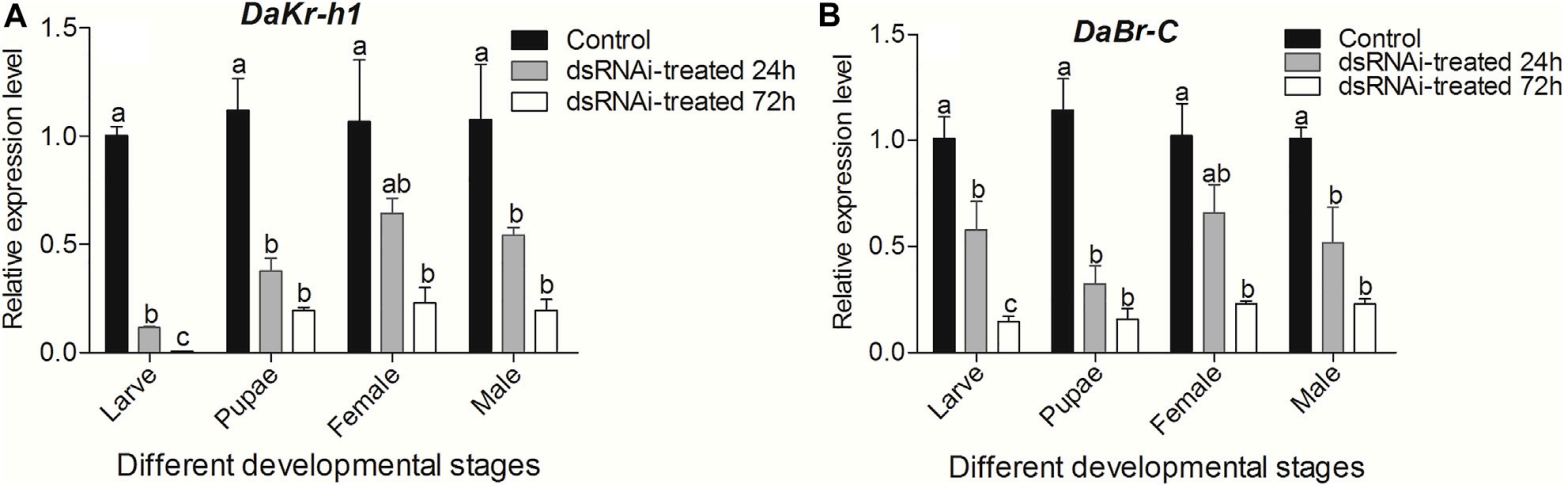
qRT-PCR analysis of *DaKr-h1* and *DaBr-C* transcript patterns in *D. armandi* after being injected with dsRNA for 24 and 72 h. **(A)** *DaKr-h1*; **(B)** *DaBr-C*. Error bars indicate standard error of the mean of three biological replicates (One-way ANOVA, *p* < 0.05, with Tukey’s test of multiple comparisons).

Analysis of *DaKr-h1* and *DaBr-C* expression after injection of ds*Kr-h1* confirmed that *DaKr-h1* and *DaBr-C* were knocked down at all developmental stages (Figure 7). Compared with the negative control and as determined by qRT-PCR, *DaKr-h1* and *DaBr-C* transcript levels at 72 h were significantly lower than those at 24 h (*p* < 0.05). These results indicate that *DaKr-h1* and *DaBr-C* gene silencing can reduce target gene expression.

#### Knockdown Effect of Injecting dsKr-h1 and dsBr-C Separately

**FIGURE 8:**
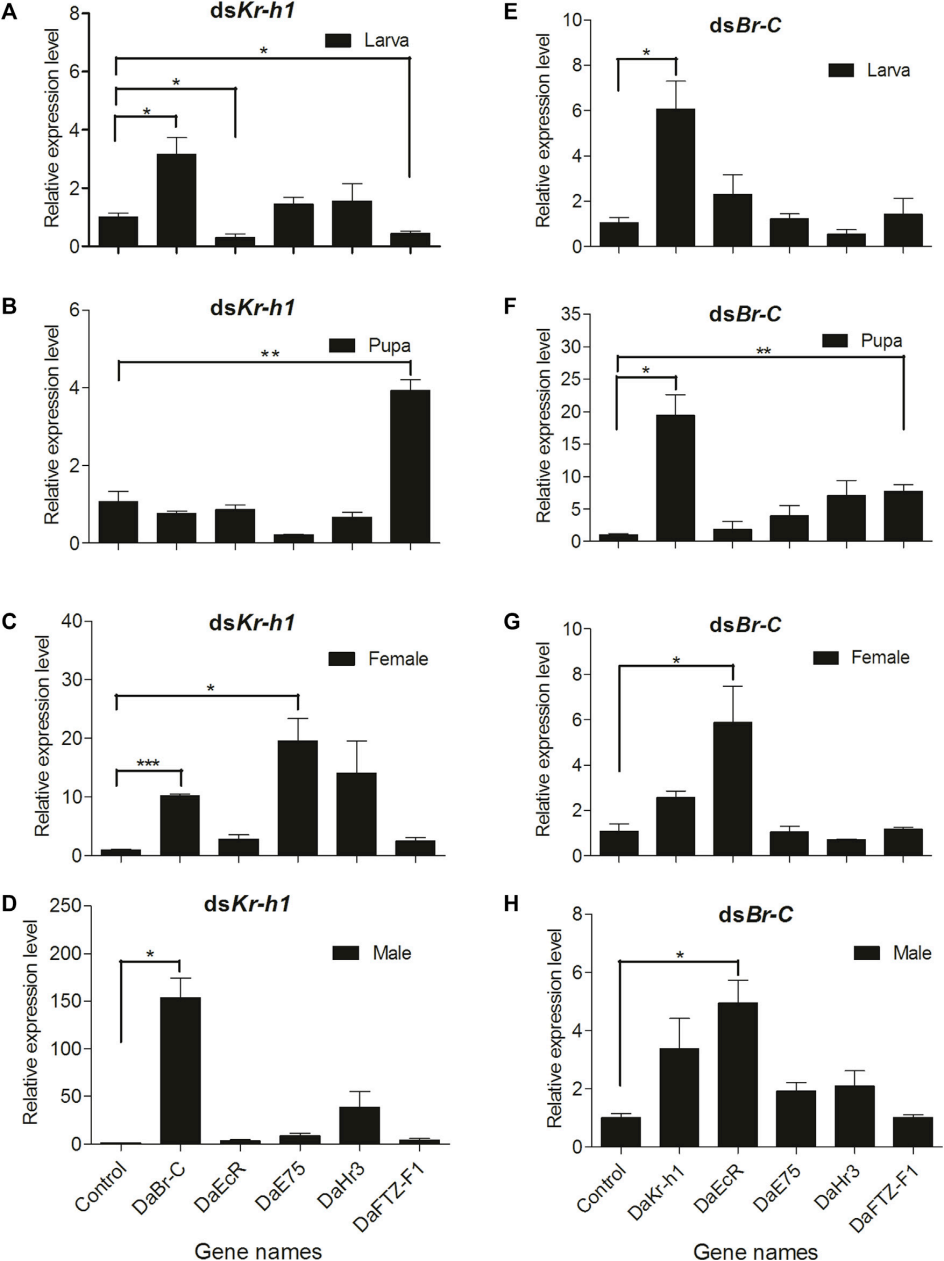
*DaKr-h1* and *DaBr-C* knockdown affects JH signaling pathway-related genes. The larvae, pupae, and emerged adults were allowed to ingest with L4440 (negative control), ds*Kr-h1*, and ds*Br-C* for 3 days. *DaKr-h1*, *DaBr-C*, *DaEcR*, *DaE75*, *DaHr3*, and *DaFTZ-F1* transcript levels were measured. *DaKr-h1* knockdown in larvae **(A)**, pupae **(B)**, adult females **(C)**, and adult males **(D)**. *DaBr-C* knockdown in larvae **(E)**, pupae **(F)**, adult females **(G)**, and adult males **(H)**. The columns represent means with vertical lines indicating the standard error. Asterisks denote significant differences (unpaired *t*-test; *p < 0.05, **p ≤ 0.01, ***p ≤ 0.001).

In this study, RNAi was used to determine *DaEcR*, *DaE75*, *DaHr3*, and *DaFTZ-F1* gene expression after *DaKr-h1* or *DaBr-C* knockdown in larval, pupal and adult stages. In the larva: *DaKr-h1* knockdown suppressed *DaEcR* and *DaFTZ-F1* expression and significantly upregulated *DaBr-C* expression but had no effect on *DaE75* and *DaHr3* expression (Figure 8A, Supplementary Table S2). *DaBr-C* knockdown significantly upregulated *DaKr-h1* expression but had no effect on *DaEcR*, *DaE75*, *DaHr3* and *DaFTZ-F1* expression (Figure 8E, Supplementary Table S2). In the pupa: *DaKr-h1* knockdown significantly upregulated *DaFTZ-F1* expression but had no effect on *DaBr-C*, *DaEcR*, *DaE75* and *DaHr3* expression (Figure 8B, Supplementary Table S2). *DaBr-C* knockdown significantly upregulated Da*Kr-h1* and suppressed *DaFTZ-F1* expression, but had no effect on *DaEcR*, *DaE75*, *DaHr3* and *DaFTZ-F1* expression (Figure 8F, Supplementary Table S2). In adult female: ds*Kr-h1* injection significantly upregulated *DaBr-C* and *DaE75* expression, with no significant effect on *DaEcR, DaHr3* and *DaFTZ-F1* expression (Figure 8C, Supplementary Table S2). After ds*Br-C* injection, *DaKr-h1* expression was significantly upregulated (Figure 8G, Supplementary Table S2). In adult male: ds*Kr-h1* injection significantly upregulated *DaBr-C* expression, whereas *DaEcR*, *DaE75*, *DaHr3* and *DaFTZ-F1* expression did not change significantly (Figure 8D, Supplementary Table S2). *DaEcR* expression was significantly upregulated after ds*Br-C* injection (Figure 8H, Supplementary Table S2).

#### Effects of dsKr-h1 and dsBr-C RNAi on the Development of *D. Armandi* Larvae and Pupae

**FIGURE 9:**
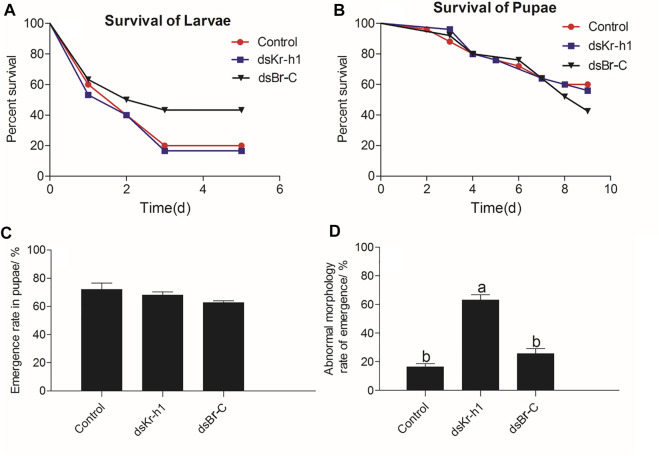
Survival, emergence, and abnormal morphology rates of *D. armandi* after RNAi. **(A)** Survival rate of larvae. **(B)** Survival rate of pupae. **(C)** Emergence rate of pupae. **(D)** Abnormal morphology rate of pupae. Each biological repetition had 25 samples. The Kaplan-Meier method (log-rank Mantel–Cox test) was used to analyze the survival rate. Data of emergence and abnormal morphology rates of pupae were analyzed by one-way ANOVA. Different letters above the bars indicate that the difference was significant at *p* < 0.05.

After larvae were treated with engineered bacteria, the survival rate of the vector (control), ds*Kr-h1*, and ds*Br-C* groups on day 5 was 20.0, 16.7, and 43.3%, respectively (Figure 9A). The Kaplan-Meier method (log-rank Mantel–Cox test) was used to analyze the survival rate. No significant difference was observed in the survival rate in larvae of the ds*Kr-h1* and ds*Br-C* groups compared with the control group (*DaKr-h1*: χ^2^ = 0.089, df = 1, *p* = 0.766; *DaBr-C*: χ^2^ = 2.404, df = 1, *p* = 0.121). After the pupae were treated with dsRNA, the survival rate of the vector (control), ds*Kr-h1*, and ds*Br-C* groups on day 9 in pupae were 60, 58, and 42.5%, respectively (Figure 9B). No significant difference was observed in the survival rate in pupae of the ds*Kr-h1* and ds*Br-C* groups compared with the control group (*DaKr-h1*: χ^2^ = 0.824, df = 1, *p* = 0.364; *DaBr-C*: χ^2^ = 0.048, df = 1, *p* = 0.826). The survival rate was 20% lower in the ds*Kr-h1* group than in the vector group (control). In addition to analyzing the larval and pupal survival rates, the effects of ds*Kr-h1* and ds*Br-C* on the emergence and abnormal morphology rates of pupae were analyzed. The emergence rate of the control, ds*Kr-h1*, and ds*Br-C* groups was 68.0, 72.0, and 62.7%, respectively. No significant difference was observed in the emergence rate of the ds*Kr-h1* and ds*Br-C* groups compared with the control group (*F =* 2.313, df = 1, *p =* 0.180; Figure 9C). The abnormal morphology rate of the ds*Kr-h1* and ds*Br-C* groups was 63.0 and 25.5% (*F =* 54.605, df = 1, *p <* 0.0001; Figure 9D). Taken together, these data suggest that *DaKr-h1* and *DaBr-C* gene silencing affects the growth and development of *D. armandi*.

#### Adult Development of dsRNA Phenotypes

**FIGURE 10:**
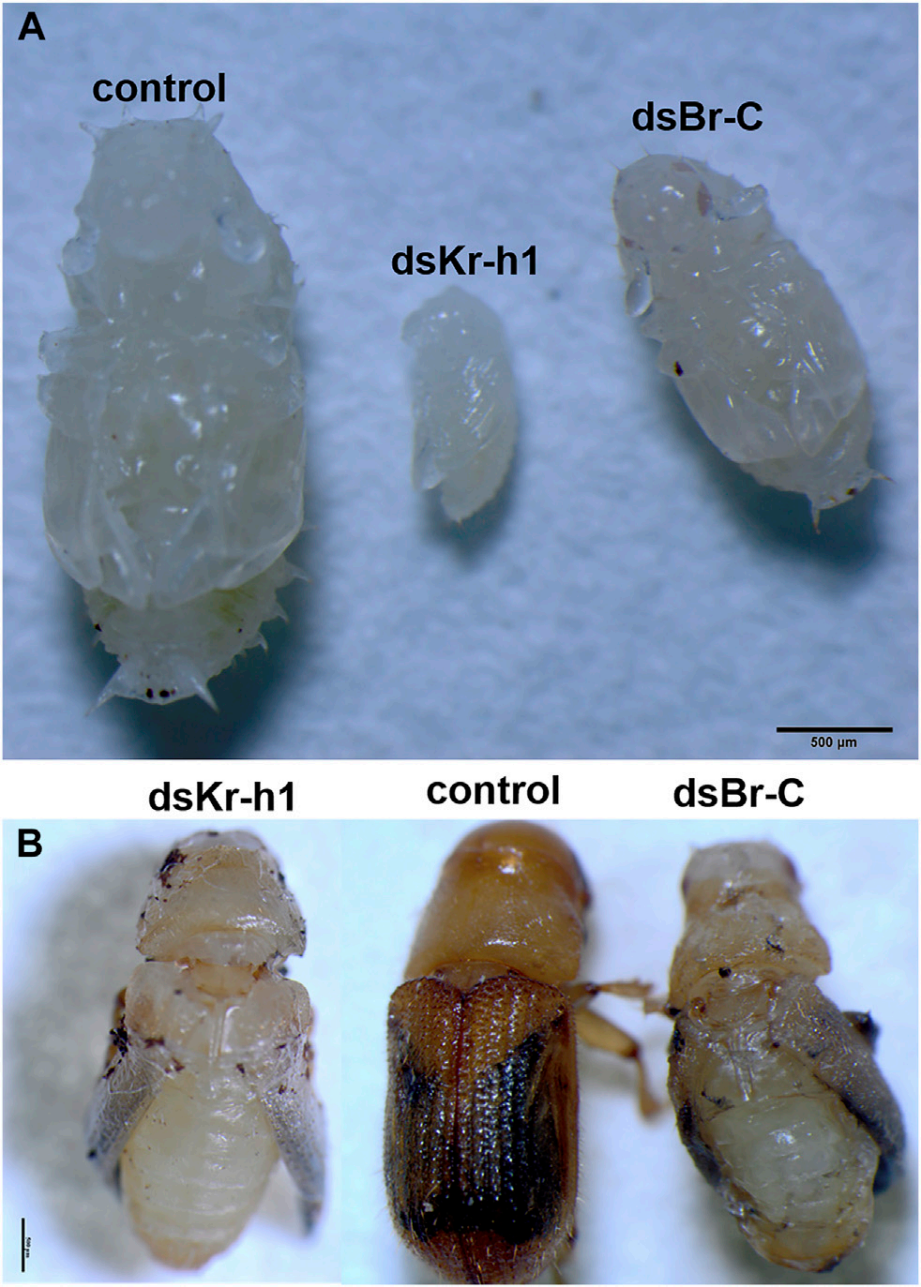
Biological morphology of *D. armandi*. **(A)** The RNAi processing larva pupation pictures; **(B)** RNAi processing pupal emergence pictures.

The phenotypes of *D. armandi* pupating larvae and adults produced by dsRNA-mediated silencing of transcripts are shown in Figure 10. *DaKr-h1* silencing resulted in early pupation of *D. armandi* in larvae (It took only 2 days for the larvae to pupate early), and the pupae were significantly smaller than the control pupae (Figure 10A). dsRNA injection of *DaKr-h1* into *D. armandi* pupae produced approximately 63% of deformed adults. Compared with the control group, the aberrant beetles had shorter carapace lengths and were neither tanned nor sclerotized (Figure 10B). Figure 10B shows the phenotypes of *D. armandi* adults produced by dsRNA-mediated transcript silencing. The injection of pupae with dsRNA for *DaBr-C* resulted in shape abnormalities in 25.5% of the treated beetles. Many parts of their appendices, including the wings and parts of the legs, were deformed or partly covered with old epicuticle.

## Discussion

In this study, we performed expressional and functional analysis of *Kr-h1* and *Br-C* identified from *D. armandi*. A phylogenetic tree constructed by aligning *DaKr-h1* and *DaBr-C* amino acid sequences with amino acid sequences from other insects showed that *DaKr-h1* and *DaBr-C* amino acid sequences cluster with known *D. ponderosae Kr-h1* and *Br-C* proteins, indicating that the identified sequences are genuine *Kr-h1* and *Br-C* orthologs of *D. armandi*.

To investigate *DaKr-h1* and *DaBr-C* expression patterns in *D. armandi*, *DaKr-h1* and *DaBr-C* expression was examined at different developmental stages. Temporal expression profiles showed that *DaKr-h1* and *DaBr-C* expression were predominantly expressed in the final larval stage (final instar, feeding larva), decreased to low levels at mature larvae (post-feeding final instar larva), and became low in the pupal stage, with expression remaining stable from the pupal to adult stage. This was similar to *Kr-h1* expression in *D. melanogaster* (Minakuchi et al., 2008), *T. castaneum* (Minakuchi et al., 2009), *Bombyx mori* (Kayukawa et al., 2014), and *Helicoverpa armigera (* *Zhang et al., 2018* *)*. The temporal expression profile of *DaBr-C* was similar to *M. sexta* (Zhou et al., 1998; Zhou and Riddiford, 2001; Zhou and Riddiford, 2002) and *T. castaneum* (Konopova and Jindra, 2008; Suzuki et al., 2008). *DaBr-C* expression was prominent during the larval–pupal transition but decreased as pupae began to develop to the adult. These results suggest that *DaKr-h1* and *DaBr-C* were essential for the metamorphosis of *D. armandi*, especially during the final larval stage.

In this study, we demonstrated that *DaKr-h1* was highly expressed in the heads of larvae and pupae. This was consistent with *kr-h1* gene expression in the brains of *D. melanogaster* larvae and *A. mellifera* worker honeybees (Grozinger et al., 2003; Shi et al., 2007). The *DaKr-h1* transcript level in adults was higher in the midgut and in the Malpighian tubules than in the head, hindgut, fat body, testes, ovaries, and antennae. Thus, the *DaKr-h1* gene exhibited a broad tissue expression pattern, reflecting a possible pleiotropic role for *Kr-h1* in *D. armandi*. Interestingly, the *DaKr-h1* gene was slightly expressed in the antennae, which are chemosensory organs bearing sensilla specialized for the detection of olfactory signals by the antennal lobes—the main olfactory center of the brain (Duportets et al., 2012).

*DaBr-C* expression was highest in the heads of larvae and pupae, and in the reproductive organs of male and female adults. *Br-C* is widely distributed in several tissues from the last larval stage of development to the pupal stage (Bayer et al., 1996; Zhou and Riddiford, 2001; Reza et al., 2004). Studies have shown that the transcription factor *Br-C* has several roles in insect oogenesis. One is the formation of the dorsal appendage of the egg chorion in *Drosophila* (Deng and Bownes, 1997; Ward and Berg, 2005). It also plays a role in the effect of nutrition on oogenesis in *Drosophila melanogaster* (Terashima and Bownes, 2005). The Broad Complex isoform 2 (BrC-Z2) transcriptional factor plays a critical role in vitellogenin transcription in the silkworm *Bombyx mori* (Yang et al., 2014). Tissue distribution studies in spotted shrimp revealed that *Br-C* was expressed in the ovaries and was higher in the ovaries than in the testes, suggesting that *Br-C* plays an important role in its reproductive development and is important in ovarian and testicular development (Buaklin et al., 2013).

JH stimulates *Kr-h1* expression in various insects (Kayukawa et al., 2012). For example, JH treatment increased *Kr-h1* expression in *Blattella germanica*, *H. armigera*, and *Nilaparvata lugens* late instar larvae (Lozano and Belles, 2011; Jin et al., 2014; Zhang et al., 2018). Interestingly, the results of the present study also showed that *DaKr-h1* expression was significantly upregulated after 20E application, which was consistent with the findings in *D. melanogaster* (Pecasse et al., 2000). *Kr-h1* expression was induced solely by JH in *T. castaneum* (Minakuchi et al., 2009), *F. occidentalis*, and *H. brevitubus* (Minakuchi et al., 2011). Furthermore, in *B. mori*, although 20E alone did not induce *Kr-h1* expression, it significantly enhanced the induction of JH, suggesting that ecdysteroids and JH act synergistically to induce *Kr-h1* expression (Kayukawa et al., 2014). Thus, the induction of *Kr-h1* in different species is complicated because of different modes of hormonal regulation. Consistent with this pattern, the data of *Pyrrhocoris apterus* show that compared to *Kr-h1*, expression of *Br-C* depends much less on JH and that, in contrast to *Kr-h1*, removal of *Br-C* cannot accelerate metamorphosis in larvae (Konopova et al., 2011). The results of the present study suggest that *DaBr-C* expression or upregulation was induced by 20E but not by JHA.

In this study, the physiological functions of *DaKr-h1* and *DaBr-C* in the metamorphosis of *D. armandi* were analyzed by RNAi. *DaKr-h1* knockdown at the larval stage of *D. armandi* caused reduced larval body weight, shorter developmental stages, and early pupation. This situation was similar to the precocious metamorphosis reported in insects such as *T. castaneum*, *Pyrrhocoris apterus*, and *B. germanica* (Minakuchi et al., 2008; Minakuchi et al., 2009; Konopova et al., 2011; Lozano and Belles, 2011). RNAi-mediated *DaKr-h1* gene silencing at the pupal stage (newly pupated pupae) promoted insect metamorphosis in the present study. In *N. lugens*, ds*Kr-h1*-treated individuals had smaller wings, and the depletion of *NlKr-h1* resulted in the partial formation of early adult features (Jin et al., 2014). Furthermore, *B. mori* with transgenes overexpressing *Kr-h1* failed to pupate, suggesting that *Kr-h1* was involved in the suppression of metamorphosis in *B. mori* (Kayukawa et al., 2014). These studies suggest that *Kr-h1* is a master repressor of insect morphogenesis. Although *Kr-h1* plays a role in suppressing insect metamorphosis in a JH-dependent manner, studies have proposed that it suppresses metamorphosis by modifying the expression of 20E-inducible genes. The *Kr-h1* protein molecule interacts with the *Br-C* gene at the *Kr-h1* binding site and suppresses *Br-C* expression in larvae (Minakuchi et al., 2009; Zhu et al., 2010; Kayukawa et al., 2016). In *D. armandi*, *DaBr-C* expression was upregulated by *DaKr-h1* knockdown in the larval stage, whereas it was downregulated by *DaKr-h1* knockdown in the pupal stage. In *Drosophila*, larvae survive until pupation after knockdown of all *Br-C* isoforms, suggesting that *Br-C* is not essential for early postembryonic development (Kiss et al., 1988). In *T. castaneum*, knockout of *Br-C* in larvae affects 20E-mediated midgut remodeling during larval-pupal metamorphosis (Parthasarathy et al., 2008). Similarly, knockdown of *Br-C* in late 4th instar *L. dispar* larvae resulted in developmental defects, *epidermis* remodelling failure, and molting disruption (Ding et al., 2020). In conclusion, *Br-C* is required for insects to complete metamorphic processes involving growth, differentiation, and tissue remodeling (Konopova and Jindra, 2008). Here, we found that *Br-C* is not essential for early postembryonic development of *D. armandi* in early larvae that survive until pupation after knockout of *Br-C*. However, knockdown of *Br-C* at the pupal stage resulted in developmental defects and wing deformities. These results suggest that *DaBr-C* plays a critical role in epidermal and wing remodeling during *D. armandi* development and molting.

After *Kr-h1* knockdown, *Br-C* expression was downregulated in the last nymphal instar of *B. germanica* (Huang et al., 2013). In the pupal stage of *T. castaneum*, exogenous JH analogs mediated *Kr-h1* upregulation and induced *Br-C* transcription (Minakuchi et al., 2009). In *M. sexta* and *B. mori*, the removal of the corpus allatum (i.e., the main organ of JH synthesis) induced *Br-C* expression and precocious metamorphosis (Zhou et al., 1998; Reza et al., 2004). Moreover, knocking down *Kr-h1* in the larval and adult stages of *D. armandi* reduced *DaEcR* expression, but knocking down *Kr-h1* in the pupal stage reduced *DaEcR*, *DaE75*, and *DaHr3* expression. In *D. melanogaster*, *Kr-h1* mutations resulted in changes in the expression patterns of ecdysone-inducible genes, such as *EcR*, *E74A*, *E75B*, *Hr3*, which together control the delayed expression of *βFTZ-F1* (King-Jones and Thummel, 2005) during the metamorphosis stage (Pecasse et al., 2000; Liu et al., 2018). These studies suggest that *Kr-h1* suppresses metamorphosis by modifying the expression of early ecdysone-inducible genes.

Based on the results of this study and previous research, it is reasonable to speculate on the hormonal regulation mechanisms of *DaKr-h1* and *DaBr-C*. *DaKr-h1* expression may be induced by JH via the Methoprene-tolerant–steroid receptor coactivator complex (Kayukawa et al., 2012; Kayukawa and Shinoda, 2015), and *DaKr-h1* molecules may subsequently repress *DaBr-C* expression. Because of JH persistence, *DaKr-h1* expression can be maintained at high levels during this stage. A decrease in JH concentration at the onset of the last instar larval stage can lead to a temporary absence of *DaKr-h1* (Kayukawa et al., 2014), contributing to the induction of *DaBr-C* by 20E during the larval–pupal transition (Reza et al., 2004; Muramatsu et al., 2008). Any apparent inconsistencies in the inter-regulatory roles of genes at different developmental stages may be due to cellular factors such as transcription factors, coactivators, repressors, promoters, epigenetic modifications, or different cellular environments, including endocrine, paracrine, and nutritional factors (Kayukawa and Shinoda, 2015).

## Data Availability

The original contributions presented in the study are included in the article/Supplementary Material, further inquiries can be directed to the corresponding author.

## References

[B1] Abdou M. A. He Q. Wen D. Zyaan O. Wang J. Xu J. (2011). *Drosophila Met* and *Gce* Are Partially Redundant in Transducing Juvenile Hormone Action. Insect Biochem. Mol. Biol. 41 (12), 938–945. 10.1016/j.ibmb.2011.09.003 21968404

[B2] Altschul S. F. Gish W. Miller W. Myers E. W. Lipman D. J. (1990). Basic Local Alignment Search Tool. J. Mol. Biol. 215 (3), 403–410. 10.1006/jmbi.1990.999910.1016/s0022-2836(05)80360-2 2231712

[B3] Bayer C. A. Holley B. Fristrom J. W. (1996). A Switch inBroad-ComplexZinc-Finger Isoform Expression Is Regulated Posttranscriptionally during the Metamorphosis ofDrosophilaImaginal Discs. Develop. Biol. 177 (1), 1–14. 10.1006/dbio.1996.0140 8660872

[B4] Bayer C. A. Kalm L. v. Fristrom J. W. (1997). Relationships between Protein Isoforms and Genetic Functions Demonstrate Functional Redundancy at theBroad-ComplexduringDrosophilaMetamorphosis. Develop. Biol. 187 (2), 267–282. 10.1006/dbio.1997.8620 9242423

[B5] Buaklin A. Sittikankaew K. Khamnamtong B. Menasveta P. Klinbunga S. (2013). Characterization and Expression Analysis of the *Broad-Complex* (*Br-C*) Gene of the Giant Tiger Shrimp *Penaeus monodon* . Comp. Biochem. Physiol. B: Biochem. Mol. Biol. 164 (4), 280–289. 10.1016/j.cbpb.2013.02.004 23485783

[B6] Chen L. Zhu J. Sun G. Raikhel A. S. (2004). The Early Gene Broad Is Involved in the Ecdysteroid Hierarchy Governing Vitellogenesis of the Mosquito *Aedes aegypti* . J. Mol. Endocrinol. 33 (3), 743–761. 10.1677/jme.1.01531 15591032

[B7] Dai L. Ma M. Wang C. Shi Q. Zhang R. Chen H. (2015). Cytochrome P450s from the Chinese white pine Beetle, *Dendroctonus Armandi* (Curculionidae: Scolytinae): Expression Profiles of Different Stages and Responses to Host Allelochemicals. Insect Biochem. Mol. Biol. 65, 35–46. 10.1016/j.ibmb.2015.08.004 26319543

[B8] Dai L. Wang C. Zhang X. Yu J. Zhang R. Chen H. (2014). Two CYP4 Genes of the Chinese white pine beetle,Dendroctonus armandi(Curculionidae: Scolytinae), and Their Transcript Levels under Different Development Stages and Treatments. Insect Mol. Biol. 23 (5), 598–610. 10.1111/imb.12108 25039485

[B9] Daimon T. Uchibori M. Nakao H. Sezutsu H. Shinoda T. (2015). Knockout Silkworms Reveal a Dispensable Role for Juvenile Hormones in Holometabolous Life Cycle. Proc. Natl. Acad. Sci. U.S.A. 112 (31), E4226. 10.1073/pnas.1506645112 26195792 PMC4534237

[B10] Deng W. M. Bownes M. (1997). Two Signalling Pathways Specify Localised Expression of the *Broad-Complex* in *Drosophila* Eggshell Patterning and Morphogenesis. Development 124 (22), 4639–4647. 10.1242/dev.124.22.4639 9409680

[B11] DiBello P. R. Withers D. A. Bayer C. A. Fristrom J. W. Guild G. M. (1991). The *Drosophila Broad-Complex* Encodes a Family of Related Proteins Containing Zinc Fingers. Genetics 129 (2), 385–397. 10.1093/genetics/129.2.385 1743483 PMC1204631

[B12] Ding N. Wang Z. Geng N. Zou H. Zhang G. Cao C. (2020). Silencing *Br-C* Impairs Larval Development and Chitin Synthesis in *Lymantria dispar* Larvae. J. Insect Physiol. 122, 104041. 10.1016/j.jinsphys.2020.104041 32126216

[B13] Duportets L. Bozzolan F. Abrieux A. Maria A. Gadenne C. Debernard S. (2012). The Transcription Factor Krüppel Homolog 1 Is Linked to the Juvenile Hormone-dependent Maturation of Sexual Behavior in the Male Moth, Agrotis Ipsilon. Gen. Comp. Endocrinol. 176 (2), 158–166. 10.1016/j.ygcen.2012.01.005 22285394

[B14] Emanuelsson O. Nielsen H. Brunak S. von Heijne G. (2000). Predicting Subcellular Localization of Proteins Based on Their N-Terminal Amino Acid Sequence. J. Mol. Biol. 300 (4), 1005–1016. 10.1006/jmbi.2000.3903 10891285

[B15] Fu D. Dai L. Gao H. Sun Y. Liu B. Chen H. (2019). Identification, Expression Patterns and RNA Interference of Aquaporins in *Dendroctonus Armandi* (Coleoptera: Scolytinae) Larvae during Overwintering. Front. Physiol. 10, 967. 10.3389/fphys.2019.00967 31427984 PMC6688586

[B16] Fu D. Dai L. Ning H. Kang X. Sun Y. Chen H. (2020). Effects of Cold Stress on Metabolic Regulation in the Overwintering Larvae of the Chinese white pine Beetle, Dendroctonus Armandi. Entomol. Exp. Appl. 168 (11), 836–850. 10.1111/eea.12991

[B17] Fu D. Sun Y. Liu B. Ning H. Wang L. Chen H. (2021). Identification, Expression Patterns and RNA Interference of Capa Peptide Receptors in *Dendroctonus Armandi* Larvae under Cold. J. Appl. Entomol. 146 (1-2), 144–157. 10.1111/jen.12941

[B18] Gasteiger E. Hoogland C. Gattiker A. Duvaud S. E. Wilkins M. R. Appel R. D. (2005). “Protein Identification And Analysis Tools On The Expasy Server,” The Proteomics Protocols Handbook. Editor Walker J. M. (Totowa, NJ: Humana Press), 571–607. 10.1385/1-59259-890-0:571

[B19] Gillespie M. J. Fisher L. (1979). Confidence Bands for the kaplan-meier Survival Curve Estimate. Ann. Statist. 7 (4), 920–924. 10.1109/SMElec.2012.6417228

[B20] Grozinger C. M. Robinson G. E. (2006). Endocrine Modulation of a Pheromone-Responsive Gene in the Honey Bee Brain. J. Comp. Physiol. A. 193 (4), 461–470. 10.1007/s00359-006-0202-x 17192826

[B21] Grozinger C. M. Sharabash N. M. Whitfield C. W. Robinson G. E. (2003). Pheromone-mediated Gene Expression in the Honey Bee Brain. Proc. Natl. Acad. Sci. U.S.A. 100 (Suppl. 2), 14519–14525. 10.1073/pnas.2335884100 14573707 PMC304112

[B22] Huang J.-H. Lozano J. Belles X. (2013). *Broad-complex* Functions in Postembryonic Development of the Cockroach *Blattella germanica* Shed New Light on the Evolution of Insect Metamorphosis. Biochim. Biophys. Acta (Bba) - Gen. Subjects 1830 (1), 2178–2187. 10.1016/j.bbagen.2012.09.025 23041750

[B23] Huang Z. Y. Lin S. Ahn K. (2016). Methoprene Does Not Affect Juvenile Hormone Titers in Honey Bee (*Apis mellifera*) Workers. Insect Sci. 25 (2), 235–240. 10.1111/1744-7917.12411 27763722

[B24] Jin M.-n. Xue J. Yao Y. Lin X.-d. (2014). Molecular Characterization and Functional Analysis of Krüppel-Homolog 1 (Kr-H1) in the Brown Planthopper, Nilaparvata Lugens (Stål). J. Integr. Agric. 13 (9), 1972–1981. 10.1016/S2095-3119(13)60654-1

[B25] Kageyama Y. Masuda S. Hirose S. Ueda H. (2003). Temporal Regulation of the Mid-prepupal Gene FTZ-F1: DHR3 Early Late Gene Product Is One of the Plural Positive Regulators. Genes to Cells 2 (9), 559–569. 10.1046/j.1365-2443.1997.1460344.x 9413997

[B26] Kayukawa T. Jouraku A. Ito Y. Shinoda T. (2017). Molecular Mechanism Underlying Juvenile Hormone-Mediated Repression of Precocious Larval-Adult Metamorphosis. Proc. Natl. Acad. Sci. U.S.A. 114 (5), 1057–1062. 10.1073/pnas.1615423114 28096379 PMC5293048

[B27] Kayukawa T. Minakuchi C. Namiki T. Togawa T. Yoshiyama M. Kamimura M. (2012). Transcriptional Regulation of Juvenile Hormone-Mediated Induction of Krüppel Homolog 1, a Repressor of Insect Metamorphosis. Proc. Natl. Acad. Sci. U.S.A. 109 (29), 11729–11734. 10.1073/pnas.1204951109 22753472 PMC3406821

[B28] Kayukawa T. Murata M. Kobayashi I. Muramatsu D. Okada C. Uchino K. (2014). Hormonal Regulation and Developmental Role of Krüppel Homolog 1, a Repressor of Metamorphosis, in the Silkworm Bombyx mori. Develop. Biol. 388 (1), 48–56. 10.1016/j.ydbio.2014.01.022 24508345

[B29] Kayukawa T. Nagamine K. Ito Y. Nishita Y. Ishikawa Y. Shinoda T. (2016). Krüppel Homolog 1 Inhibits Insect Metamorphosis via Direct Transcriptional Repression of Broad-Complex, a Pupal Specifier Gene. J. Biol. Chem. 291 (4), 1751–1762. 10.1074/jbc.M115.686121 26518872 PMC4722455

[B30] Kayukawa T. Shinoda T. (2015). Functional Characterization of Two Paralogous JH Receptors, Methoprene-Tolerant 1 and 2, in the Silkworm, *Bombyx mori* (Lepidoptera: Bombycidae). Appl. Entomol. Zool. 50 (3), 383–391. 10.1007/s13355-015-0345-8

[B31] King-Jones K. Thummel C. S. (2005). Nuclear Receptors - a Perspective from *Drosophila* . Nat. Rev. Genet. 6 (4), 311–323. 10.1038/nrg1581 15803199

[B32] Kiss I. Beaton A. H. Tardiff J. Fristrom D. Fristrom J. W. (1988). Interactions and Developmental Effects of Mutations in the *Broad-Complex* of *Drosophila melanogaster* . Genetics 118 (2), 247–259. 10.1093/genetics/118.2.247 3129334 PMC1203278

[B33] Kiss I. Bencze G. Fodor A. Szabad J. Fristrom J. W. (1976). Prepupal Larval Mosaics in *Drosophila melanogaster* . Nature 262 (5564), 136–138. 10.1038/262136a0 819843

[B34] Konopova B. Jindra M. (2008). *Broad-Complex* Acts Downstream of *Met* in Juvenile Hormone Signaling to Coordinate Primitive Holometabolan Metamorphosis. Development 135 (3), 559–568. 10.1242/dev.016097 18171683

[B35] Konopova B. Smykal V. Jindra M. (2011). Common and Distinct Roles of Juvenile Hormone Signaling Genes in Metamorphosis of Holometabolous and Hemimetabolous Insects. Plos One 6 (12), e28728. 10.1371/journal.pone.0028728 22174880 PMC3234286

[B36] Kumar S. Stecher G. Tamura K. (2016). MEGA7: Molecular Evolutionary Genetics Analysis Version 7.0 for Bigger Datasets. Mol. Biol. Evol. 33 (7), 1870–1874. 10.1093/molbev/msw054 27004904 PMC8210823

[B37] Lam G. Hall B. L. Bender M. Thummel C. S. (1999). DHR3 Is Required for the Prepupal-Pupal Transition and Differentiation of Adult Structures during Drosophila Metamorphosis. Develop. Biol. 212 (1), 204–216. 10.1006/dbio.1999.9343 10419696

[B38] Le S. Q. Gascuel O. (2008). An Improved General Amino Acid Replacement Matrix. Mol. Biol. Evol. 25 (7), 1307–1320. 10.1093/molbev/msn067 18367465

[B39] Li Z. Dai L. Chu H. Fu D. Sun Y. Chen H. (2018). Identification, Expression Patterns, and Functional Characterization of Chemosensory Proteins in *Dendroctonus Armandi* (Coleoptera: Curculionidae: Scolytinae). Front. Physiol. 9, 291. 10.3389/fphys.2018.00291 29636701 PMC5881420

[B40] Liu B. Fu D. Gao H. Ning H. Sun Y. Chen H. (2021). Cloning and Expression of the Neuropeptide F and Neuropeptide F Receptor Genes and Their Regulation of Food Intake in the Chinese white pine Beetle *Dendroctonus Armandi* . Front. Physiol. 12, 662651. 10.3389/fphys.2021.662651 34220532 PMC8249871

[B41] Liu S. Li K. Gao Y. Liu X. Chen W. Ge W. (2018). Antagonistic Actions of Juvenile Hormone and 20-hydroxyecdysone within the Ring Gland Determine Developmental Transitions in *Drosophila* . Proc. Natl. Acad. Sci. U.S.A. 115 (1), 139–144. 10.1073/pnas.1716897115 29255055 PMC5776822

[B42] Lozano J. Belles X. (2011). Conserved Repressive Function of Krüppel Homolog 1 on Insect Metamorphosis in Hemimetabolous and Holometabolous Species. Sci. Rep. 1 (1), 163. 10.1038/srep00163 22355678 PMC3240953

[B43] Minakuchi C. Namiki T. Shinoda T. (2009). Krüppel Homolog 1, an Early Juvenile Hormone-Response Gene Downstream of Methoprene-Tolerant, Mediates its Anti-metamorphic Action in the Red Flour Beetle Tribolium castaneum. Develop. Biol. 325 (2), 341–350. 10.1016/j.ydbio.2008.10.016 19013451

[B44] Minakuchi C. Namiki T. Yoshiyama M. Shinoda T. (2008). RNAi-Mediated Knockdown of Juvenile Hormone Acid O-Methyltransferase Gene Causes Precocious Metamorphosis in the Red Flour Beetle *Tribolium castaneum* . Febs J. 275 (11), 2919–2931. 10.1111/j.1742-4658.2008.06428.x 18435763

[B45] Minakuchi C. Tanaka M. Miura K. Tanaka T. (2011). Developmental Profile and Hormonal Regulation of the Transcription Factors Broad and Krüppel Homolog 1 in Hemimetabolous Thrips. Insect Biochem. Mol. Biol. 41 (2), 125–134. 10.1016/j.ibmb.2010.11.004 21111817

[B46] Minakuchi C. Zhou X. Riddiford L. M. (2008). Krüppel Homolog 1 (Kr-H1) Mediates Juvenile Hormone Action during Metamorphosis of Drosophila melanogaster. Mech. Develop. 125, 91–105. 10.1016/j.mod.2007.10.002 PMC2276646 18036785

[B47] Muramatsu D. Kinjoh T. Shinoda T. Hiruma K. (2008). The Role of 20-hydroxyecdysone and Juvenile Hormone in Pupal Commitment of the Epidermis of the Silkworm, *Bombyx mori* . Mech. Develop. 125 (5-6), 411–420. 10.1016/j.mod.2008.02.001 18331786

[B48] Parthasarathy R. Tan A. Bai H. Palli S. R. (2008). Transcription Factor Broad Suppresses Precocious Development of Adult Structures during Larval-Pupal Metamorphosis in the Red Flour Beetle, Tribolium castaneum. Mech. Develop. 125 (3-4), 299–313. 10.1016/j.mod.2007.11.001 PMC3556786 18083350

[B49] Parvy J.-P. Wang P. Garrido D. Maria A. Blais C. Poidevin M. (2014). Forward and Feedback Regulation of Cyclic Steroid Production in *Drosophila melanogaster* . Development 141 (20), 3955–3965. 10.1242/dev.102020 25252945

[B50] Pecasse F. Beck Y. Ruiz C. Richards G. (2000). Krüppel-homolog, a Stage-specific Modulator of the Prepupal Ecdysone Response, Is Essential for Drosophila Metamorphosis. Develop. Biol. 221 (1), 53–67. 10.1006/dbio.2000.9687 10772791

[B51] Reza A. M. S. Kanamori Y. Shinoda T. Shimura S. Mita K. Nakahara Y. (2004). Hormonal Control of a Metamorphosis-specific Transcriptional Factor *Broad-Complex* in Silkworm. Comp. Biochem. Physiol. Part B: Biochem. Mol. Biol. 139 (4), 753–761. 10.1016/j.cbpc.2004.09.009 15581808

[B52] Riddiford L. M. (1996). Juvenile Hormone: The Status of its “Status Quo” Action. Arch. Insect Biochem. Physiol. 32 (3-4), 271–286. 10.1002/(SICI)1520-6327(1996)32:3/4<271:AID-ARCH2>3.0.CO;2-W 8756300

[B53] Riddiford L. M. (1994). Cellular and Molecular Actions of Juvenile Hormone I. General Considerations and Premetamorphic Actions. Adv. Insect Physiol. 24, 213–274. 10.1016/S0065-2806(08)60084-3

[B54] Riddiford L. M. Cherbas P. Truman J. W. (2000). Ecdysone Receptors and Their Biological Actions. Vitam Horm. 60, 1–73. 10.1016/s0083-6729(00)60016-x 11037621

[B55] Riddiford L. M. (2012). How Does Juvenile Hormone Control Insect Metamorphosis and Reproduction? Gen. Comp. Endocrinol. 179 (3), 477–484. 10.1016/j.ygcen.2012.06.001 22728566

[B56] Riddiford L. M. Truman J. W. Mirth C. K. Shen Y.-c. (2010). A Role for Juvenile Hormone in the Prepupal Development of *Drosophila melanogaster* . Development 137 (7), 1117–1126. 10.1242/dev.037218 20181742 PMC2835327

[B57] Shi L. Lin S. Grinberg Y. Beck Y. Grozinger C. M. Robinson G. E. (2007). Roles ofDrosophila Kruppel-Homolog 1 in Neuronal Morphogenesis. Devel Neurobio 67 (12), 1614–1626. 10.1002/dneu.20537 17562531

[B58] Smykal V. Daimon T. Kayukawa T. Takaki K. Shinoda T. Jindra M. (2014). Importance of Juvenile Hormone Signaling Arises with Competence of Insect Larvae to Metamorphose. Develop. Biol. 390 (2), 221–230. 10.1016/j.ydbio.2014.03.006 24662045

[B59] Sun Y. Fu D. Kang X. Liu B. Ning H. Chen H. (2021). Function of Mevalonate Pathway Genes in the Synthesis of Frontalin in Chinese white pine Beetle, *Dendroctonus Armandi* (Curculionidae: Scolytinae). Arch. Insect Biochem. Physiol. 107 (4), e21828. 10.1002/arch.21828 34173689

[B60] Sun Y. Fu D. Liu B. Wang L. Chen H. (2022). Functional Characterization of Allatostatin C (PISCF/AST) and Juvenile Hormone Acid O-Methyltransferase in *Dendroctonus Armandi* . Ijms 23 (5), 2749. 10.3390/ijms23052749.c 35269892 PMC8910878

[B61] Suzuki Y. Truman J. W. Riddiford L. M. (2008). The Role of Broad in the Development ofTribolium Castaneum:implications for the Evolution of the Holometabolous Insect Pupa. Development 135 (3), 569–577. 10.1242/dev.015263 18171684

[B62] Terashima J. Bownes M. (2005). A Microarray Analysis of Genes Involved in Relating Egg Production to Nutritional Intake in *Drosophila melanogaster* . Cell Death Differ 12 (5), 429–440. 10.1038/sj.cdd.4401587 15776001

[B63] Ureña E. Chafino S. Manjón C. Franch-Marro X. Martín D. (2016). The Occurrence of the Holometabolous Pupal Stage Requires the Interaction between E93, Krüppel-Homolog 1 and Broad-Complex. Plos. Genet. 12 (5), e1006020. 10.1371/journal.pgen.1006020 27135810 PMC4852927

[B64] Ureña E. Manjón C. Franch-Marro X. Martín D. (2014). Transcription Factor *E93* Specifies Adult Metamorphosis in Hemimetabolous and Holometabolous Insects. Proc. Natl. Acad. Sci. U.S.A. 111 (19), 7024–7029. 10.1073/pnas.1401478111 24778249 PMC4024875

[B65] Ward E. J. Berg C. A. (2005). Juxtaposition between Two Cell Types Is Necessary for Dorsal Appendage Tube Formation. Mech. Develop. 122 (2), 241–255. 10.1016/j.mod.2004.10.006 15652711

[B66] White K. P. Hurban P. Watanabe T. Hogness D. S. (1997). Coordination of *Drosophila Metamorphosis* by Two Ecdysone-Induced Nuclear Receptors. Science 276 (5309), 114–117. 10.1126/science.276.5309.114 9082981

[B67] Yang C. Lin Y. Liu H. Shen G. Luo J. Zhang H. (2014). The *Broad Complex* Isoform 2 (*BrC-Z2*) Transcriptional Factor Plays a Critical Role in Vitellogenin Transcription in the Silkworm *Bombyx mori* . Biochim. Biophys. Acta (Bba) - Gen. Subjects 1840 (9), 2674–2684. 10.1016/j.bbagen.2014.05.013 24861733

[B68] Zhang R. Gao G. Chen H. (2016). Silencing of the Olfactory Co-receptor Gene in *Dendroctonus Armandi* Leads to EAG Response Declining to Major Host Volatiles. Sci. Rep. 6 (1), 23136. 10.1038/srep23136 26979566 PMC4793246

[B69] Zhang W.-N. Ma L. Liu C. Chen L. Xiao H.-J. Liang G.-M. (2018). Dissecting the Role ofKrüppel Homolog 1in the Metamorphosis and Female Reproduction of the Cotton bollworm,Helicoverpa Armigera. Insect Mol. Biol. 27 (4), 492–504. 10.1111/imb.12389 29719076

[B70] Zhang Z. Xu J. Sheng Z. Sui Y. Palli S. R. (2011). Steroid Receptor Co-activator Is Required for Juvenile Hormone Signal Transduction through a bHLH-PAS Transcription Factor, Methoprene Tolerant. J. Biol. Chem. 286 (10), 8437–8447. 10.1074/jbc.M110.191684 21190938 PMC3048728

[B71] Zhao M. Dai L. Fu D. Gao J. Chen H. (2017). Electrophysiological and Behavioral Responses of *Dendroctonus Armandi* (Coleoptera: Curculionidae: Scolytinae) to Two Candidate Pheromone Components: Frontalin and Exo-Brevicomin. Chemoecology 27 (3), 91–99. 10.1007/s00049-017-0235-3

[B72] Zhou B. Hiruma K. Shinoda T. Riddiford L. M. (1998). Juvenile Hormone Prevents Ecdysteroid-Induced Expression of *Broad Complex* RNAs in the Epidermis of the Tobacco Hornworm,*Manduca Sexta* . Develop. Biol. 203 (2), 233–244. 10.1006/dbio.1998.9059 9808776

[B73] Zhou B. Riddiford L. M. (2001). Hormonal Regulation and Patterning of the *Broad-Complex* in the Epidermis and wing Discs of the Tobacco Hornworm, *Manduca Sexta* . Develop. Biol. 231 (1), 125–137. 10.1006/dbio.2000.0143 11180957

[B74] Zhou X. Riddiford L. M. (2002). Broad Specifies Pupal Development and Mediates the 'Status Quo' Action of Juvenile Hormone on the Pupal-Adult Transformation inDrosophilaandManduca. Development 129 (9), 2259–2269. 10.1242/dev.129.9.2259 11959833

[B75] Zhu J. Busche J. M. Zhang X. (2010). Identification of Juvenile Hormone Target Genes in the Adult Female Mosquitoes. Insect Biochem. Mol. Biol. 40 (1), 23–29. 10.1016/j.ibmb.2009.12.004 20018242

