# Supplementary figures and images for: Roles of Krüppel Homolog 1 and Broad-Complex in the Development of Dendroctonus armandi (Coleoptera: Scolytinae)
Source: Front Physiol. 2022 Apr 6;13:865442. doi: 10.3389/fphys.2022.865442 (PMC9019567; doi:10.3389/fphys.2022.865442)

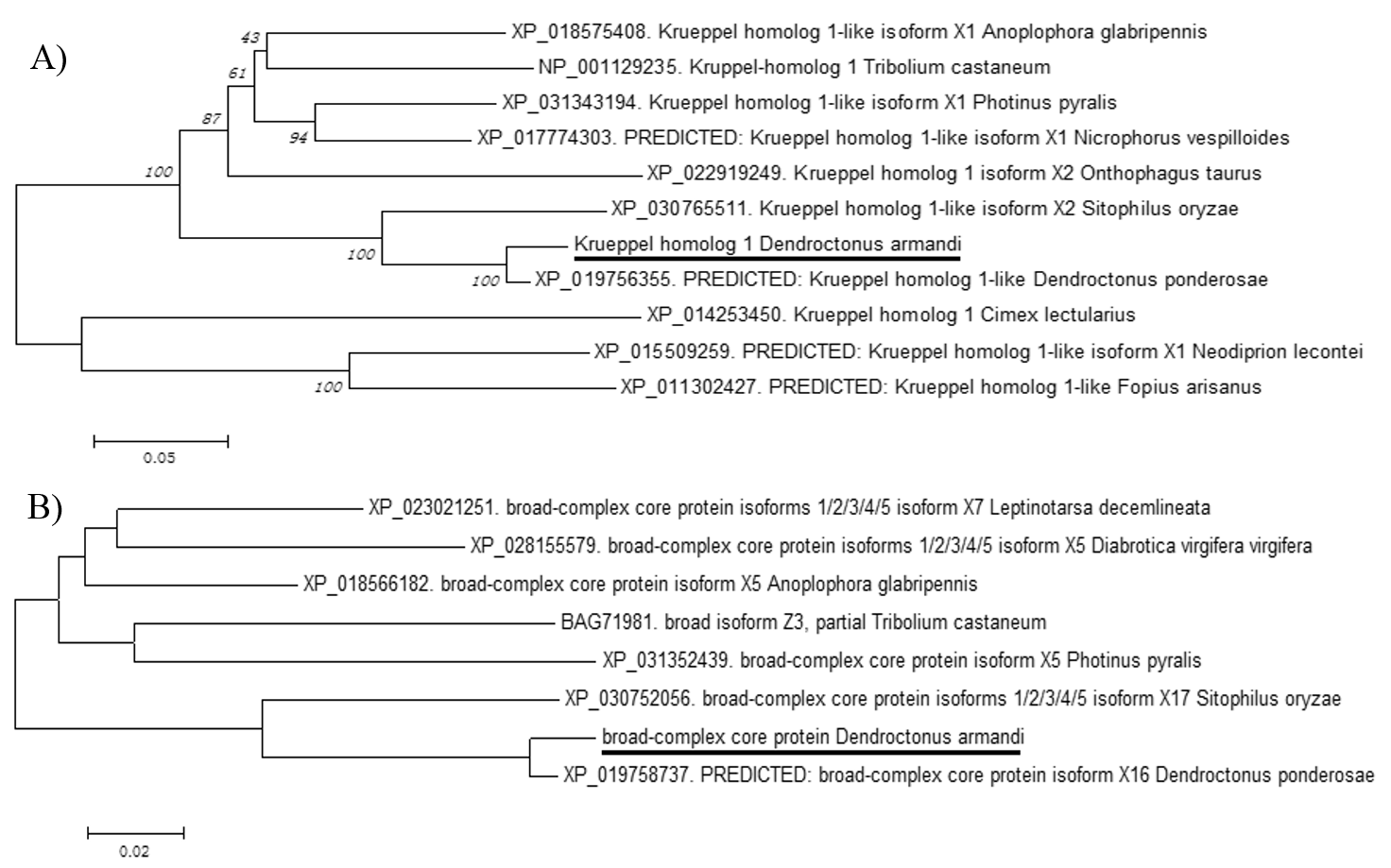

Supplement: Supplementary file 1 [file Image1.TIF]
