# Supplementary material for: Roles of Krüppel Homolog 1 and Broad-Complex in the Development of Dendroctonus armandi (Coleoptera: Scolytinae)
Source: Front Physiol. 2022 Apr 6;13:865442. doi: 10.3389/fphys.2022.865442 (PMC9019567; doi:10.3389/fphys.2022.865442)
Supplement: Supplementary file 2 [file DataSheet1.docx]

**Table S1**. Primers designed by Primer Primier 5.0 used in the research.

| Genes name | Primer direction (5 ‘-3’) | | Application | TM (°C) | Amplification efficiency (%) |
| --- | --- | --- | --- | --- | --- |
| *DaKr-h1* | *F* | TATCTTCAGACCTCCTATCC | cDNA amplification |  |  |
|  | *R* | ACTGTATGACGCTGTATCG |  |  |  |
| *DaBRC* | *F* | CCATTCCCAATCATCAAGG | cDNA amplification |  |  |
|  | *R* | TGGAGAAGGCGAACAAGGT |  |  |  |
| *DaKr-h1* | *Inner* | GCCAGCGAAACCAGCGACTT | 3’ RACE |  |  |
|  | *Outer* | GCGACGACATCAGAAAGC |  |  |  |
| *DaBRC* | *Inner* | CCATTCCCAATCATCAAGG | 3’ RACE |  |  |
|  | *Outer* | ATCTATCTCACAATCCGCCTCA |  |  |  |
| *DaKr-h1* | *Inner* | GCGTCATAATGCGCTCTTCT | 5’ RACE |  |  |
|  | *Outer* | CCCGAGGGCGAGTAGAAGT |  |  |  |
| *DaBRC* | *Inner* | TTCTTTCAAATCGTTCCCTCCT | 5’ RACE |  |  |
|  | *Outer* | GATGGTGGTGAAGTGAATAAGG |  |  |  |
| *DaKr-h1* | *F* | TGCGAGTTCTGCGACAA | RT-qPCR | 58.3 | 102.5 |
|  | *R* | AAACGGTGCATTTGTGC |  |  |  |
| *DaBRC* | *F* | ATCTATCTCACAATCCGCCTCA | RT-qPCR | 60 | 90.1 |
|  | *R* | CGATCTGTGCTGCTTATTCCA |  |  |  |
| *DaECR* | *F* | CGAGTGAGGTAATGATGTT | RT-qPCR | 57.4 | 95.8 |
|  | *R* | GACAATGGCAGTGAGTAA |  |  |  |
| *DaFTZ-F1* | *F* | TAGCACTTGGACAGGTAA | RT-qPCR | 57.4 | 99.2 |
|  | *R* | TTGAACTGGAAGGACTTG |  |  |  |
| *DaE75* | *F* | CAAACCGCCAGCATGATCTGT | RT-qPCR | 60 | 98 |
|  | *R* | CAGCCCGATTTCCGCATCCG |  |  |  |
| *DaHr3* | *F* | TTACGGCGTCATCACCT | RT-qPCR | 60 | 93.9 |
|  | *R* | GACTCATTCCCAACCTG |  |  |  |
| *CYP4G55* | *F* | ATGGCTTTCTTGGATCTCCT | RT-qPCR | 60 | 100.2 |
|  | *R* | GCAGTCGTATCGTGACCCTC |  |  |  |
| *β-actin* | *F* | CATCAGGAAGGACTTGTA | RT-qPCR | 60 |  |
|  | *R* | GATTCGTCGTATTCTTGTT |  |  |  |
| *DaKr-h1* | *F* | TGCTCTAGAGCAGCGAGTTCTGCGACAAGG | dsRNA synthesis |  |  |
|  | *R* | TCCCCCGGGGGAGCGAAATATGCGGGATGG |  |  |  |
| *DaBRC* | *F* | TGCTCTAGAGCACCATTCCCAATCATCAAGG | dsRNA synthesis |  |  |
|  | *R* | TCCCCCGGGGGATGGAGAAGGCGAACAAGGT |  |  |  |

**Note**: Xba 1 and Sma 1 endonuclease sequences are underlined

**Table S2** Unpaired t-test of 6 JH signaling pathway-related genes expression levels after knocking down *DaKr-h1* or *DaBr-C* from different development stages of *D. armandi.*

| **RNAi Group** | **Developmental Stage** | **Gene Name** | **t** | **p** |
| --- | --- | --- | --- | --- |
| dsKr-h1 | Larva | *DaKr-h1* | - | - |
|  |  | *DaBr-C* | -3.556 | **0.024** |
|  |  | *DaECR* | 4.133 | **0.014** |
|  |  | *DaE75* | -1.67 | 0.17 |
|  |  | *DaHr3* | -0.913 | 0.45 |
|  |  | *DaFTZ-F1* | 3.802 | **0.019** |
|  | Pupa | *DaKr-h1* | - | - |
|  |  | *DaBr-C* | 1.14 | 0.318 |
|  |  | *DaECR* | 0.718 | 0.513 |
|  |  | *DaE75* | 3.287 | 0.081 |
|  |  | *DaHr3* | 1.406 | 0.232 |
|  |  | *DaFTZ-F1* | -7.737 | **0.002** |
|  | Female | *DaKr-h1* | - | - |
|  |  | *DaBr-C* | -29.794 | **0.001** |
|  |  | *DaECR* | -2.621 | 0.117 |
|  |  | *DaE75* | -4.864 | **0.04** |
|  |  | *DaHr3* | -2.388 | 0.139 |
|  |  | *DaFTZ-F1* | -2.504 | 0.126 |
|  | Male | *DaKr-h1* | - | - |
|  |  | *DaBr-C* | -7.406 | **0.018** |
|  |  | *DaECR* | -3.051 | 0.079 |
|  |  | *DaE75* | -2.839 | 0.103 |
|  |  | *DaHr3* | -2.287 | 0.149 |
|  |  | *DaFTZ-F1* | -1.798 | 0.147 |
| dsBr-C | Larva | *DaKr-h1* | -4.001 | **0.016** |
|  |  | *DaBr-C* | - | - |
|  |  | *DaECR* | -1.405 | 0.233 |
|  |  | *DaE75* | -0.554 | 0.609 |
|  |  | *DaHr3* | 1.677 | 0.169 |
|  |  | *DaFTZ-F1* | -0.494 | 0.648 |
|  | Pupa | *DaKr-h1* | -5.764 | **0.028** |
|  |  | *DaBr-C* | - | - |
|  |  | *DaECR* | -0.757 | 0.524 |
|  |  | *DaE75* | -1.962 | 0.121 |
|  |  | *DaHr3* | -2.658 | 0.057 |
|  |  | *DaFTZ-F1* | -6.339 | **0.003** |
|  | Female | *DaKr-h1* | -3.489 | **0.025** |
|  |  | *DaBr-C* | - | - |
|  |  | *DaECR* | -2.922 | 0.092 |
|  |  | *DaE75* | 0.079 | 0.941 |
|  |  | *DaHr3* | 1.227 | 0.287 |
|  |  | *DaFTZ-F1* | -0.284 | 0.79 |
|  | Male | *DaKr-h1* | -2.207 | 0.092 |
|  |  | *DaBr-C* | - | - |
|  |  | *DaECR* | -4.991 | **0.034** |
|  |  | *DaE75* | -2.706 | 0.054 |
|  |  | *DaHr3* | -2.008 | 0.115 |
|  |  | *DaFTZ-F1* | 0.015 | 0.989 |

**Note**: P < 0.05 are marked in bold

**Figure. S1** Phylogenetic tree of *DaKr-h1* and *DaBr-C* from *D. armandi*, the two sequences from *D. armandi* were marked with black. (A): *DaKr-h1*, (B): *DaBr-C*. The phylogenetic trees were constructed with MEGA7.0, using the neighbor-joining method. Values indicated at the nodes are bootstrap values based on 1000 replicates.

**
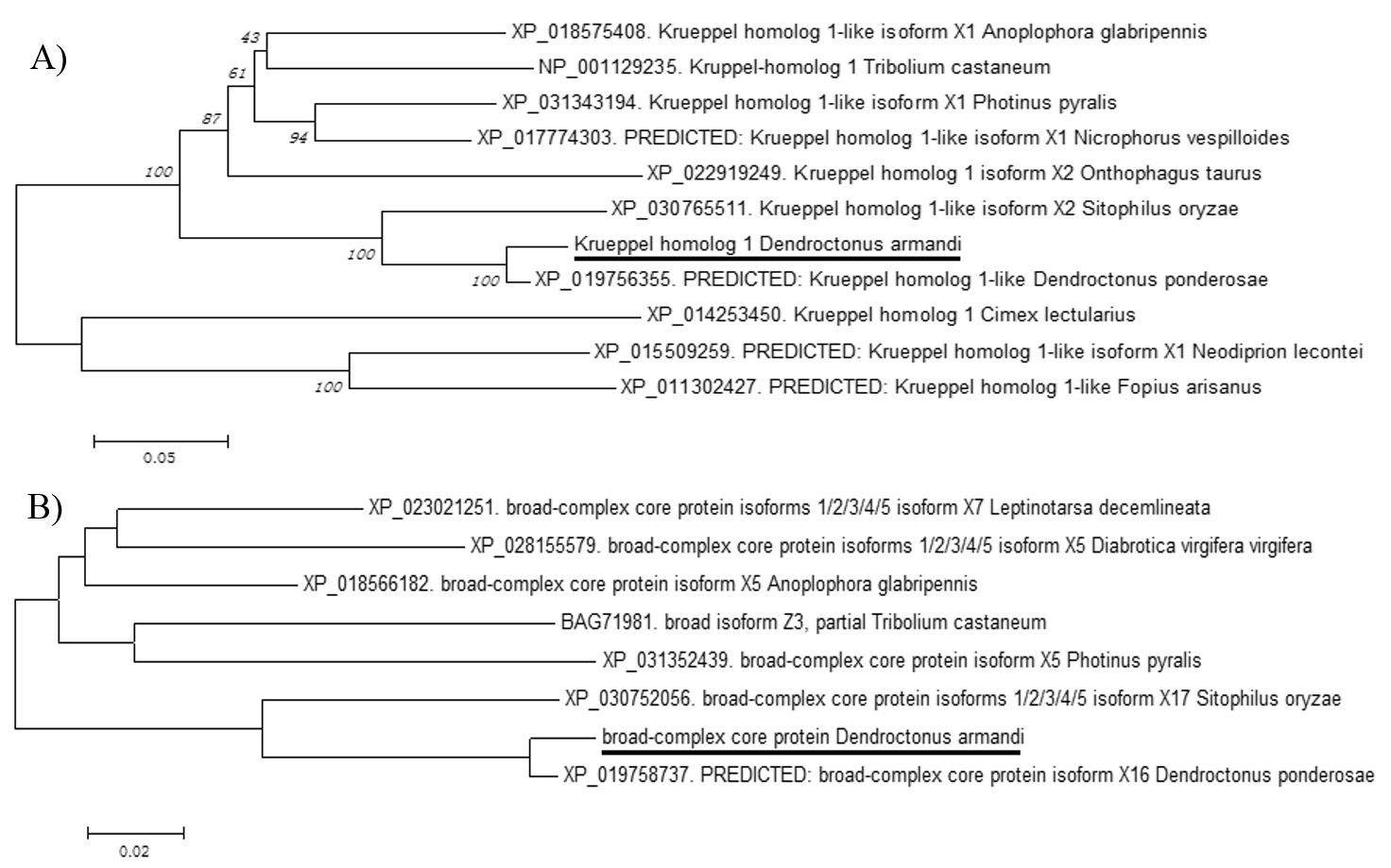
**
